# Comparative Analysis of the Microbial Community Structures Between Healthy and Anthracnose-Infected Strawberry Rhizosphere Soils Using Illumina Sequencing Technology in Yunnan Province, Southwest of China

**DOI:** 10.3389/fmicb.2022.881450

**Published:** 2022-05-16

**Authors:** Daifa Su, Shanyan Chen, Wenxing Zhou, Junyu Yang, Zhiwei Luo, Zhenrong Zhang, Yunxia Tian, Qionge Dong, Xuemei Shen, Shijie Wei, Jiangyun Tong, Xiaolong Cui

**Affiliations:** ^1^Yunnan Institute of Microbiology, School of Life Sciences, Yunnan University, Kunming, China; ^2^State Key Laboratory for Conservation and Utilization of Bio-Resources in Yunnan, Yunnan University, Kunming, China; ^3^Kunming Academy of Agricultural Science, Kunming, China

**Keywords:** strawberry anthracnose, rhizosphere soil, microbial community, high-throughput sequencing technology, cultivated strawberry

## Abstract

Anthracnose caused by *Colletotrichum* spp. was widespread in recent years and resulted in great damage to strawberry production. Soil microbial communities were key contributors to host nutrition, development, and immunity; however, the difference between the microbial communities of healthy and anthracnose-infected strawberry rhizosphere soils remains unclear. In this study, the Illumina sequencing technique was used to comparatively study the prokaryotic and fungal community compositions and structures between healthy and anthracnose-infected strawberry rhizosphere soils in Yuxi, Yunnan Province. Both microbial community diversities and richness of anthracnose-infected strawberry rhizosphere soils were higher than those of healthy strawberry rhizosphere soils. A total of 2,518 prokaryotic and 556 fungal operational taxonomic units (OTUs) were obtained at the 97% similarity threshold. Proteobacteria, Thaumarchaeota, and Acidobacteria were the dominant prokaryotic phyla; Ascomycota, unclassified_k__Fungi, and Mortierellomycota were the dominant fungal phyla. The relative abundances of beneficial bacterial phyla Actinobacteria and Firmicutes, genera *Streptomyces, Azospirillum*, and *Bacillus* were significantly reduced in anthracnose-infected strawberry rhizosphere soils; the relative abundance of beneficial fungal species *Trichoderma asperellum* shows a similar tendency with bacterial abundance. Besides *Colletotrichum*, 15 other potential fungal pathogen genera and seven fungal pathogen species were identified; among the potential pathogen genera and species, eight pathogen genera and *Fusarium oxysporum* showed significant differences between healthy and anthracnose-infected strawberry rhizosphere soils. The results suggested that strawberry planted in this area may be infected by other fungal pathogens except for *Colletotrichum* spp. Our present research will provide theoretical basis and data reference for the isolation and identification of strawberry pathogens and potential probiotics in future works.

## Introduction

Cultivated strawberry (*Fragaria* × *ananassa* Duch.) is one of the most popular fruits around the world (Cheng et al., 2014). Since it originated in the eighteenth century, cultivated strawberries have been broadly introduced all over the world (Su et al., 2018) and thousands of varieties have been developed for commercial purposes. Strawberry plants, like other commercial crops, can be damaged by environmental, genetic, and biological factors, either directly or by interactions between these factors (Garrido et al., 2011). In the field, strawberry plants can be infected by a large number of pathogens, including fungi, bacteria, viruses, nematodes, and arthropods, and these pathogens cause damage to the leaves, roots, crowns, and fruits (Husaini and Neri, 2016), resulting in the greatest fruit losses.

*Colletotrichum* spp. comprises a diverse range of important phytopathogenic fungi that cause pre- and postharvest crop losses all over the world. Many important economic crops, such as peach, chili, almond, citrus, apple, blueberry, tomato, dragon fruits, and mango, can be infected by *Colletotrichum* spp. (Peres et al., 2005; Phoulivong et al., 2010; Liao et al., 2012; Zakaria, 2021). Since Brooks (1931) first reported strawberry anthracnose in 1931, the disease has been reported all over the world; in China, strawberry anthracnose was first reported in 1990 by Hu (1990). The species *C. acutatum, C. fragariae*, and *C. gloeosporioides* have been reported as the major causal agents of strawberry anthracnose (Karimi et al., 2015; Husaini and Neri, 2016; Reddy, 2016). All three species can be found in all parts of the strawberry plant (Denoyes-Rothan et al., 2003), including fruits, flowers, leaves, runners, roots, and crowns; the typical symptoms are described as irregular and black leaf spots, flower blight, and fruit and crown rot (Freeman and Katan, 1997; Mertely and Legard, 2004). Other studies reported that species, including *C. aenigma, C. boninense, C. changpingense, C. dematium, C. foriniae, C. fructicola, C. godetiae, C. kahawae, C. karstii, C. miaoliense, C. nymphaeae, C. salicis, C. siamense, C. simmondsii, C. theobromicola, C. truncatum*, and *C. viniferum* (Mass, 1978; Singh et al., 2003; Bi et al., 2017; Adhikari et al., 2019; Dai et al., 2019; Hoseini et al., 2019; Liu et al., 2021), and *Glomerella cingulate* (Marian et al., 2020), can also cause strawberry anthracnose. The American Phytopathological Society (APS) also reported that *Gloeosporium* spp. is the pathogen of strawberry anthracnose (https://www.apsnet.org/edcenter/resources/commonnames/Pages/Strawberry.aspx).

The rhizosphere, the area surrounding plant roots (Li et al., 2020), consisting of soil, bacteria, archaea, eukaryotic microorganisms, and viruses, is one of the most complex microbial habitats on earth (Bowya and Balachandar, 2020; Li et al., 2021a), which plays a key role in nutrient acquisition, enhanced stress tolerance, protection against soil-borne pathogens, and host immune regulation (Pérez-Jaramillo et al., 2016; Pantigoso et al., 2020; Sun et al., 2020). Scientists have developed a wide range of methods to study microbial diversities and community structures in rhizosphere soils, including culture-dependent approaches (Garrido et al., 2008), Biolog microplates (Choi and Dobbs, 1999), DGGE (denaturing gradient gel electrophoresis) (Lu et al., 2012), and PLFA (phospholipid fatty acid) method (Yao et al., 2016). However, due to the complex components in soil samples, it is hard to isolate and cultivate the microorganisms from soil samples (approximately 1% can get the pure cultures) (Amann et al., 1995; Roose-Amsaleg et al., 2001). Developing new methods to study complex rhizosphere microbial diversities and community structures is urgent. With the advance of high-throughput sequencing techniques, making it possible to understand uncultured microorganisms in the rhizosphere soil of plants, it has been applied to study the complexity of microbial communities, and achievements have been made in the research of rhizosphere soil microorganisms using high-throughput sequencing technologies (Wang et al., 2019; Zhao et al., 2019; Yang et al., 2020b; Zhou et al., 2020).

China is the biggest strawberry planting country, and strawberry production has been ranked first in the world since 1994 (http://www.fao.org/faostat/zh/#data/QC/visualize). After Hu (1990) first reported strawberry anthracnose in China, Chinese researchers have done work on the strawberry anthracnose pathogens and found that *C. acutatum* is the first reported strawberry anthracnose pathogen (Dai et al., 2006), the main pathogens causing anthracnose of strawberry in China include *C. acutatum, C. fragariae*, and *C. gloeosporioides*, and the dominant species of strawberry anthracnose in different regions are varied (Jayawardena et al., 2016). Yunnan Province, located in the southwest of China, with diverse and complex landforms and climates as its main geographical advantages, is rich in abundant agricultural and sideline products. Strawberry cultivation started in the early 1980s in Yunnan Province, and now Yunnan is the only province in which strawberry cultivation can be carried out all year round in China with open-air cultivation. Until now, the microbial community differences between healthy and anthracnose-infected strawberry rhizosphere soils still remain unknown in Yunnan Province. In this study, a high-throughput sequencing technique was used to comparatively study the prokaryotic and fungal community differences between healthy and anthracnose-infected strawberry rhizosphere soils.

## Materials and Methods

### Sample Collection

The experiment was carried out at an open field in Hongta District of Yuxi City (102.547871 E, 24.450693 N, 1651.0 ± 16.69 m), Yunnan Province, China. In this experiment, an open-field, strawberry cultivar Akihime has been cultivated using rotation with scallion (*Allium fistulosum* L. var. *gigantum* Makino) for 4 years. On November 16, 2018, samples were collected from the field in Hongta District using a five-point sampling method in the flowering and fruit ripening stages. Healthy (h) and disease-infected strawberry plants (d) were chosen, the whole plants were uprooted after removing the dead branches and leaves from the soil surface, and the rhizosphere soil samples were collected after strongly shaking the plants and brushing soil from root surfaces (Desgarennes et al., 2014; Edwards et al., 2015; Coleman-Derr et al., 2016; Cui et al., 2019). Each sample consisted of five subsamples, and healthy and anthracnose-infected strawberry plant samples were composed of three replicate samples, respectively. Healthy strawberry rhizosphere soil samples were marked as hRZ (hRZ 1–3, three replicates), and diseased strawberry rhizosphere soil samples were marked as dRZ (dRZ 1–3, three replicates). All samples were kept in ice bags, transported to laboratory, and immediately stored at −80°C for extraction of genomic DNA.

### Total DNA Extraction, PCR Amplification, and Illumina Sequencing

The total genomic DNA of soil samples was directly extracted using the PowerSoil DNA Isolation Kit (Mo Bio Laboratories, San Diego, CA, USA), following the manufacturer's protocol. The concentration and purity of the extracted DNA were measured using a NanoDrop 2000 spectrometer (Thermo Fisher Scientific, Wilmington, DE, USA).

The prokaryotic universal primer pairs 341F (5′-CCTAYGGGRBGCASCAG-3′) and 806R (5′-GGACTACNNGGGTATCTAAT-3′) were used to amplify the V3–V4 region of 16S rRNA gene (Yu et al., 2010; Yang et al., 2020a), and the fungi primer pairs ITS3F (5′-GCATCGATGAAGAACGCAGC-3′) and ITS4R (5′-TCCTCCGCTTATTGATATGC-3′) were used to amplify the ITS2 region (Tedersoo et al., 2015; Yang et al., 2020a). PCR amplification was conducted using TransGen AP221-02: TransStart FastPfu DNA Polymerase (TransGen Biotech, Beijing, China) and performed in a GeneAmp 9700 thermal cycler (Applied Biosystems, Foster City, CA, USA). The reaction mixture included 4 μL 5 × FastPfu buffer, 2 μL 2.5 mM dNTPs, 0.4 μL FastPfu polymerase, 0.8 μL each primer (5 mM), 0.2 μL BSA, 10 ng template DNA, and ddH_2_O to a final volume of 20 μL. Thermal cycling conditions were as follows: 95°C for 3 min; V3–V4 region was followed by 27 cycles of 95°C for 30 s, 53°C for 30 s, and 72°C for 45 s; ITS2 region was followed by 35 cycles of 95°C for 30 s, 55°C for 30 s, and 72°C for 45 s, with a final extension at 72°C for 10 min. PCR amplification was detected using 2% agarose gel electrophoresis. The V3–V4 region and ITS2 region were sequenced on the Illumina MiSeq PE 300 platform at Shanghai Majorbio Bio-Pharm Technology Co., Ltd., following the manufacturer's protocols.

### Data Analysis

Since the data from the MiSeq sequencing platform were paired-end sequences, the paired reads were joined, quality controlled, and filtered to obtain high-quality reads using FLASH (v.1.2.11) (Magoč and Salzberg, 2011) and Trimmomatic (v.0.38) (Bolger et al., 2014) software. The obtained high-quality reads were processed to generate operational taxonomic units (OTUs) at a 97% sequence similarity threshold (Edgar, 2013). The taxonomic assignments were performed by the RDP classifier algorithm (http://rdp.cme.msu.edu/) against the Unite (v.8.0) ITS database (Abarenkov et al., 2010) and SILVA (SSU 132) 16S rRNA database (Quast et al., 2013) with a confidence threshold of 70%. All sequences taxonomically assigned to chloroplasts and mitochondria were removed in this study.

Bioinformatics analysis was performed on the free I-Sanger platform (Majorbio Bio-Pharm Technology Co. Ltd., Shanghai, China; www.i-sanger.com). Alpha diversity, which reflects the richness and diversity of microbial communities, including Sobs, Shannon, Simpson, Ace, Chao, and Coverage indexes, was calculated by Mothur (v.1.35.1; https://www.mothur.org/). Beta diversity was estimated as a representation of the compositional differences between communities, and NMDS (non-metric multidimensional scaling) was used to evaluate similarities across community structures using the Bray–Curtis distance metric. The potential functions of the prokaryotic and fungal communities between healthy and diseased rhizosphere soils were predicted by PICRUSt2 (Phylogenetic Investigation of Communities by Reconstruction of Unobserved States) (Douglas et al., 2020) and FunGuild (Fungi Functional Guild) (Nguyen et al., 2016) programs, respectively.

The data were collected using Microsoft Excel 2013, statistical analysis was performed using SPSS 17.0 (SPSS Inc., Chicago, IL, USA), the results were represented as the means ± SD (standard deviations), and *P* <0.05 was statistically significant.

## Results

### General Characteristics of Illumina MiSeq Sequencing-Based 16S rRNA and ITS Datasets

In this study, six samples were collected and processed for high-throughput sequencing and analysis. A total of 227,697 and 302,804 raw reads were produced by the Illumina MiSeq PE 300 sequencing platform for prokaryotes and fungi; after low-quality reads were filtered, a total of 192,585 and 290,950 clean reads were obtained for prokaryotes and fungi, respectively (Supplementary Table 1). The reads in fungal samples were higher than in prokaryotic samples to obtain more informatics from samples. OTU numbers in healthy samples were significantly lower than in diseased samples for the prokaryotic community; for the fungal community, the tendency was similar to the prokaryotic community but did not show a significant difference (Supplementary Table 1). The good coverage in each sample was more than 98%, suggesting that the sequencing in each sample in this study was sufficient for subsequent analyses (Supplementary Table 1).

### Richness and Diversity of Prokaryotic and Fungal Communities in Different Samples

In this study, microbial community richness and diversity were assessed using alpha and beta diversity analyses. At the 97% threshold, the rarefaction curves of prokaryotic and fungal communities were nearly reached asymptotes, indicating that the sequence depth in each sample was sufficient, and most of the prokaryotic and fungal community diversities of the samples were captured (Supplementary Figure 1).

The community diversity was expressed by Shannon and Simpson indices; community richness was expressed by Ace, Chao, and Sobs indices. Shannon indices and community richness showed similar trends; the values in healthy samples were lower than in diseased samples (Table 1). Simpson index in this study showed an opposite tendency with the Shannon index (Table 1).

**Table 1:** Alpha diversity of soil samples.

**Amplification region**	**Sample names**	**Community diversity**		**Community richness**		
		**Shannon**	**Simpson**	**Ace**	**Chao**	**Sobs**
16S	hRZ	5.164448 ± 0.159077a	0.040018 ± 0.011942a	1926.902 ± 38.777a	1934.752 ± 21.968a	1479.67 ± 32.470a
	dRZ	5.668405 ± 0.316821a	0.030974 ± 0.022890a	2256.683 ± 56.521b	2248.021 ± 81.013b	1814.67 ± 49.095b
ITS2	hRZ	3.144065 ± 0.033529a	0.121094 ± 0.003328a	431.615 ± 43.173a	402.605 ± 18.402a	322.00 ± 4.583a
	dRZ	3.221678 ± 0.028277a	0.110033 ± 0.003182a	486.477 ± 64.739a	452.106 ± 9.757a	352.00 ± 13.115a

*hRZ, healthy rhizosphere soils; dRZ, diseased rhizosphere soils (M ± SD, n = 3).*

*P-values are based on matched samples t-test. Significant values (P <0.05) are denoted by different small letters*.

**Figure 1:**
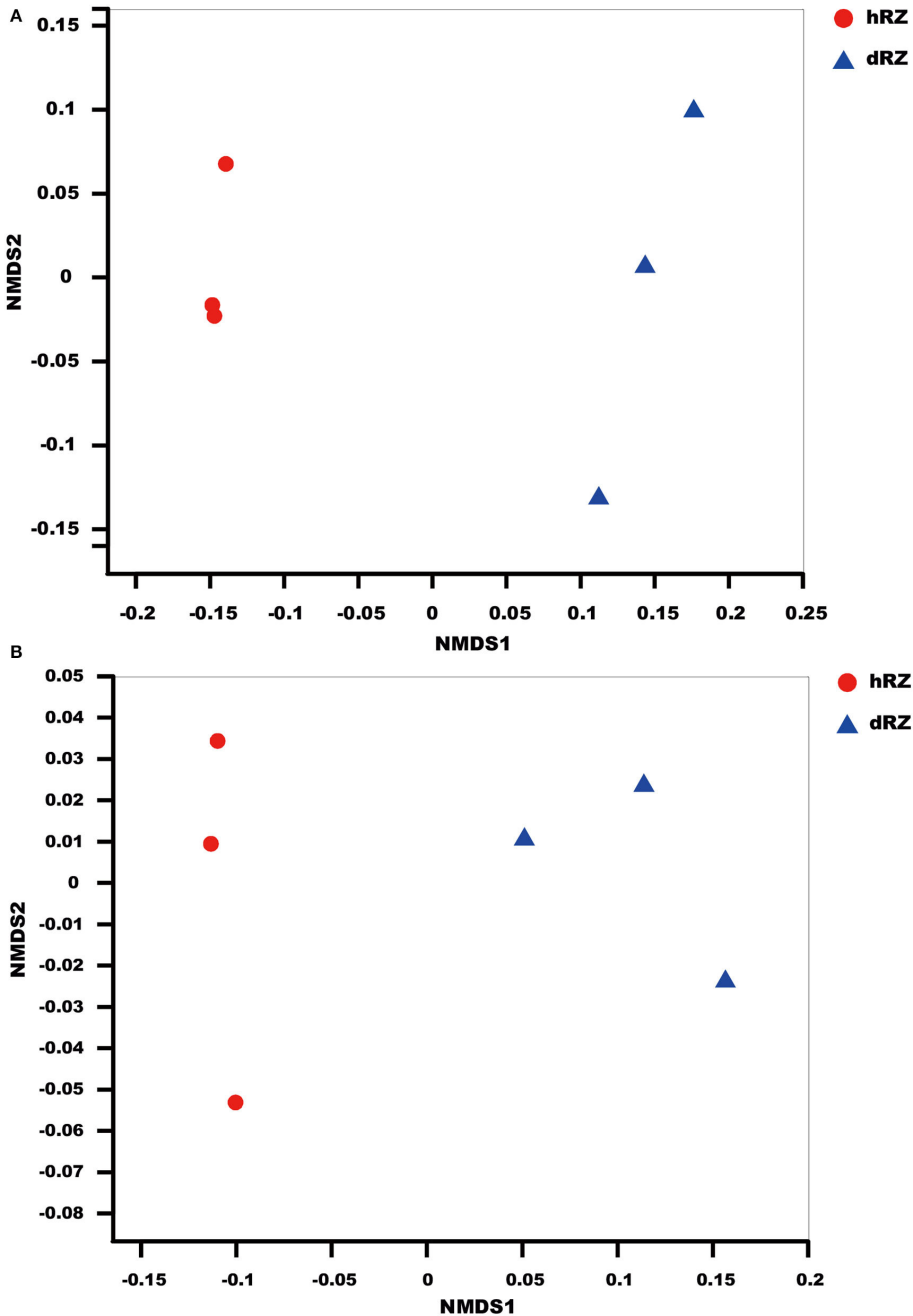
NMDS (non-metric multidimensional scaling) ordination based on Bray–Curtis similarities of prokaryotic **(A)** and fungal **(B)** OTU community structures among different samples. hRZ, healthy rhizosphere soils; dRZ, diseased rhizosphere soils (*n* = 3).

NMDS results indicated that the soil samples were divided into different groups for both prokaryotic and fungal communities and showed a good ordination (stress <0.1) (Figure 1).

### Prokaryotic and Fungal Community Composition

**Figure 2:**
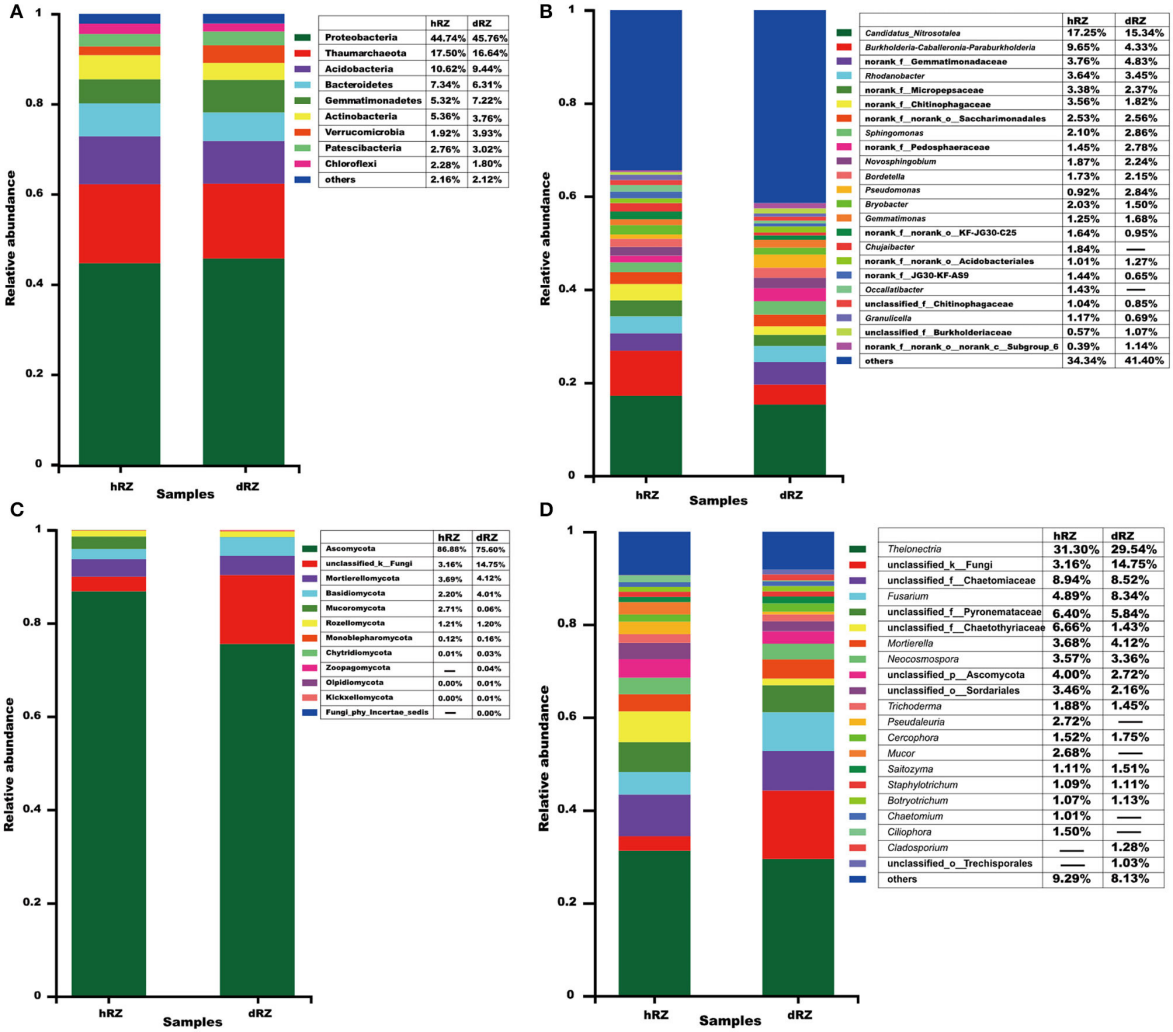
Relative abundances of prokaryotic **(A,B)** and fungal **(C,D)** community compositions at the phylum **(A,C)** and genus **(B,D)** levels are determined in different samples. The relative abundance <1% is combined into “others.” hRZ, healthy rhizosphere soils; dRZ, diseased rhizosphere soils (*n* = 3).

For the prokaryotic community, a total of 27 phyla, 62 classes, 180 orders, 309 families, 609 genera, and 2,518 OTUs were identified from six rhizosphere soil samples; 12 phyla, 30 classes, 63 orders, 122 families, 196 genera, and 556 OTUs were identified from six rhizosphere soil samples for the fungal community. At the phylum level, at least nine prokaryotic phyla showed their relative abundances >1%, and Proteobacteria was the most abundant phylum in both healthy and diseased rhizosphere soils (Figure 2A); for fungus, 10 phyla were identified from healthy rhizosphere soils, 12 phyla were identified from diseased rhizosphere soils, and Ascomycota was the most abundant phylum in both healthy and diseased rhizosphere soils (Figure 2C).

At the genus level, 23 prokaryotic genera were identified with relative abundance >1%, and the most abundant prokaryotic genera, including *Candidatus*_*Nitrosotalea, Burkholderia*–*Caballeronia*–*Paraburkholderia, Rhodanobacter, Sphingomonas, Novosphingobium, Bordetella, Pseudomonas, Bryobacter, Gemmatimonas, Chujaibacter, Occallatibacter, Granulicella*, and no rank and unclassified groups; hRZ and dRZ had 20 and 17 prokaryotic genera with a relative abundance >1%, respectively (Figure 2B). Twenty-one fungal genera showed that relative abundance > 1%, and the most abundant fungal genera, including *Thelonectria, Fusarium, Mortierella, Neocosmospora, Trichoderma, Pseudaleuria, Cercophora, Mucor, Saitozyma, Staphylotrichum, Botryotrichum, Chaetomium, Ciliophora, Cladosporium*, and unclassified groups; 19 and 17 fungal genera showed that relative abundance >1% in hRZ and dRZ, respectively (Figure 2D).

**Figure 3:**
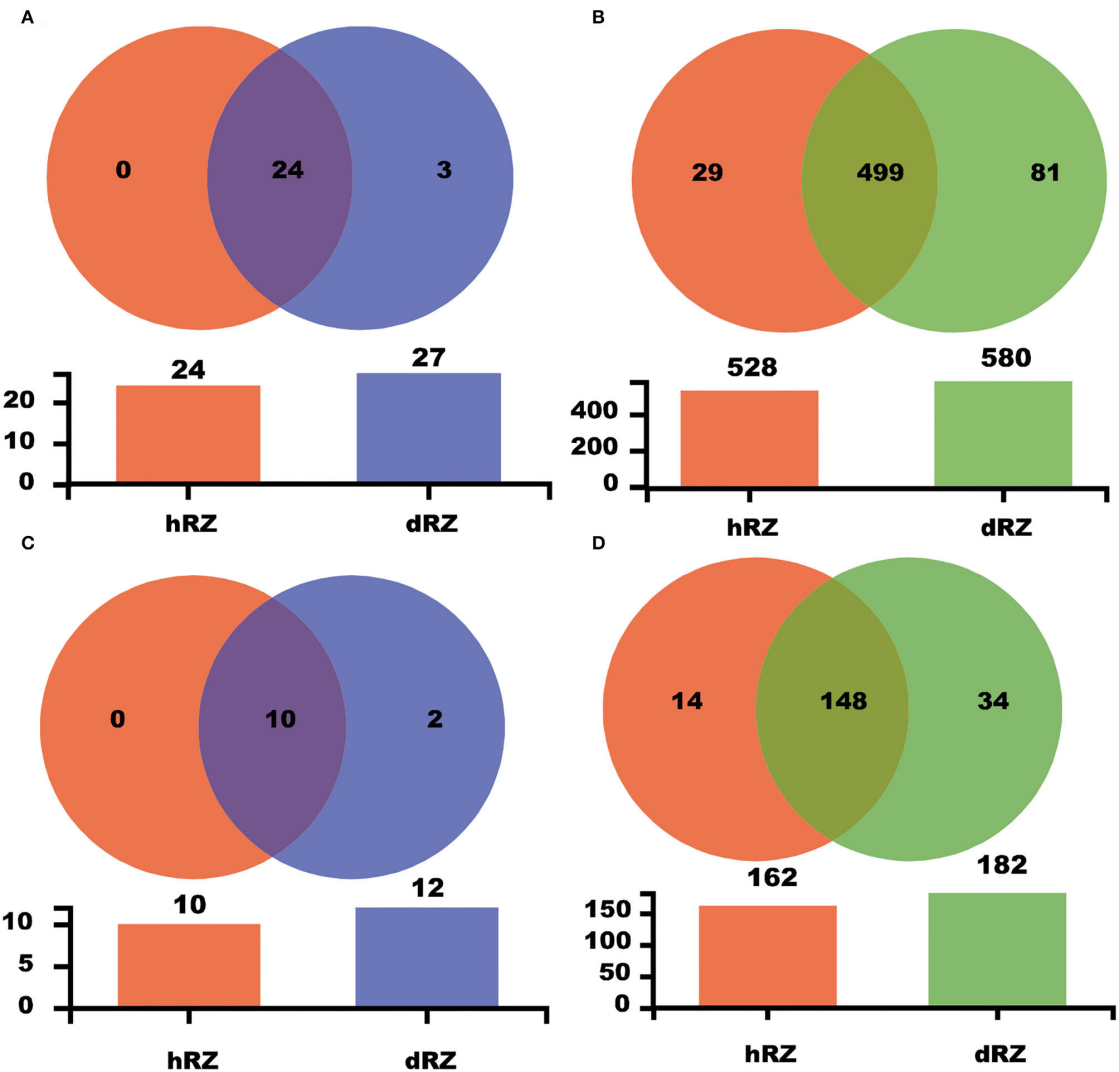
Venn diagram showing the common and unique OTUs among different samples at the phylum **(A,C)** and genus **(B,D)** levels for prokaryotic **(A,B)** and fungal **(C,D)** communities, respectively. hRZ, healthy rhizosphere soils; dRZ, diseased rhizosphere soils (*n* = 3).

Common and unique taxonomic groups in two soil samples were visualized with a Venn diagram. At the phylum level, 24 prokaryotic phyla, namely, Proteobacteria, Diapherotrites, Actinobacteria, FCPU426, Firmicutes, Latescibacteria, Nitrospirae, Dependentiae, Acidobacteria, Planctomycetes, Euryarchaeota, Thaumarchaeota, Cyanobacteria, Armatimonadetes, Spirochaetes, Bacteroidetes, WPS-2, Gemmatimonadetes, Elusimicrobia, Verrucomicrobia, Chloroflexi, Rokubacteria, Patescibacteria, and unclassified_k__norank_d__Bacteria that were contained in both hRZ and dRZ; Epsilonbacteraeota, WS2, and Zixibacteria, were the prokaryotic phyla that were contained only in dRZ (Figure 3A and Supplementary Figure 2). Ten fungal phyla, namely, Mortierellomycota, Chytridiomycota, Ascomycota, Mucoromycota, Monoblepharomycota, Basidiomycota, Rozellomycota, Olpidiomycota, Kickxellomycota, and unclassified_k__Fungi, were contained in both hRZ and dRZ. Zoopagomycota and Fungi_phy_Incertae_sedis were the fungal phyla that were contained only in dRZ (Figures 2C, 3C and Supplementary Figure 3). At the genus level, 499 prokaryotic genera were contained in both hRZ and dRZ and 29 and 81 prokaryotic genera were contained only in hRZ and dRZ, respectively (Figure 3B and Supplementary Figure 4). One hundred and forty-eight fungal genera were contained in both hRZ and dRZ, and 14 and 34 fungal genera were contained only in hRZ and dRZ, respectively (Figure 3D and Supplementary Figure 5).

### Comparative Analysis of Two Soil Groups

**Figure 4:**
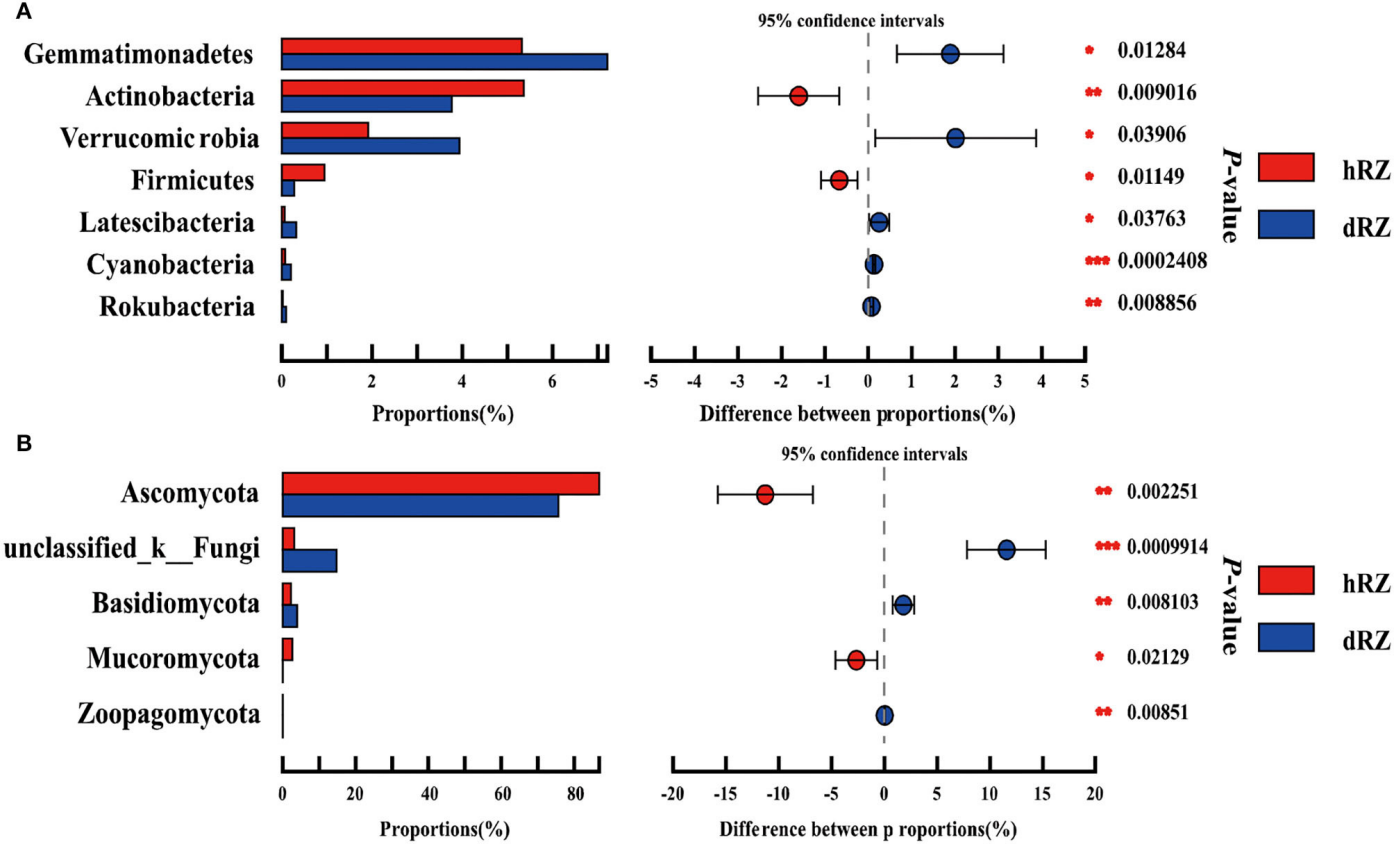
Relative abundances of prokaryotic **(A)** and fungal **(B)** phyla show significant differences between healthy and diseased rhizosphere soils. *Indicates significantly different values, *0.01 < *P* ≤ 0.05, **0.001 < *P* ≤ 0.01, ***P ≤ 0.001. *P*-values are based on Student's *t*-tests. hRZ, healthy rhizosphere soils; dRZ, diseased rhizosphere soils (*n* = 3).

Comparative analysis was used to study the prokaryotic and fungal community differences at the phylum and genus levels between healthy and diseased rhizosphere soils. Among 27 prokaryotic phyla, seven phyla were significantly different between healthy and diseased rhizosphere soils, the relative abundances of Rokubacteria, Cyanobacteria, Latescibacteria, Verrucomicrobia, and Gemmatimonadetes were significantly enriched in diseased rhizosphere soils compared with healthy rhizosphere soils, while the relative abundances of potential beneficial phyla Actinobacteria and Firmicutes in diseased rhizosphere soils were significantly reduced compared with healthy rhizosphere soils (Figure 4A). Among 12 fungal phyla, five phyla showed significant differences between healthy and diseased rhizosphere soils, the relative abundances of Ascomycota and Mucoromycota in healthy rhizosphere soils were significantly higher than in diseased rhizosphere soils, and the relative abundances of Basidiomycota and Zoopagomycota showed an opposite tendency with Ascomycota and Mucoromycota (Figure 4B).

Among 609 identified prokaryotic genera in this study, 190 genera showed significant differences between healthy and diseased rhizosphere soils (data not shown). Among 190 genera with significant differences, 134 genera showed that the relative abundances in diseased rhizosphere soils were significantly higher than in healthy rhizosphere soils, while the other 56 genera showed an opposite tendency with the 134 genera. A total of 52 fungal genera showed significant differences between healthy and diseased rhizosphere soils, 26 genera showed that the relative abundances in healthy rhizosphere soils were significantly higher than in diseased rhizosphere soils, and the other 26 genera showed an opposite tendency (data not shown).

### Abundance of Pathogenic and Beneficial Species in Different Samples

**Figure 5:**
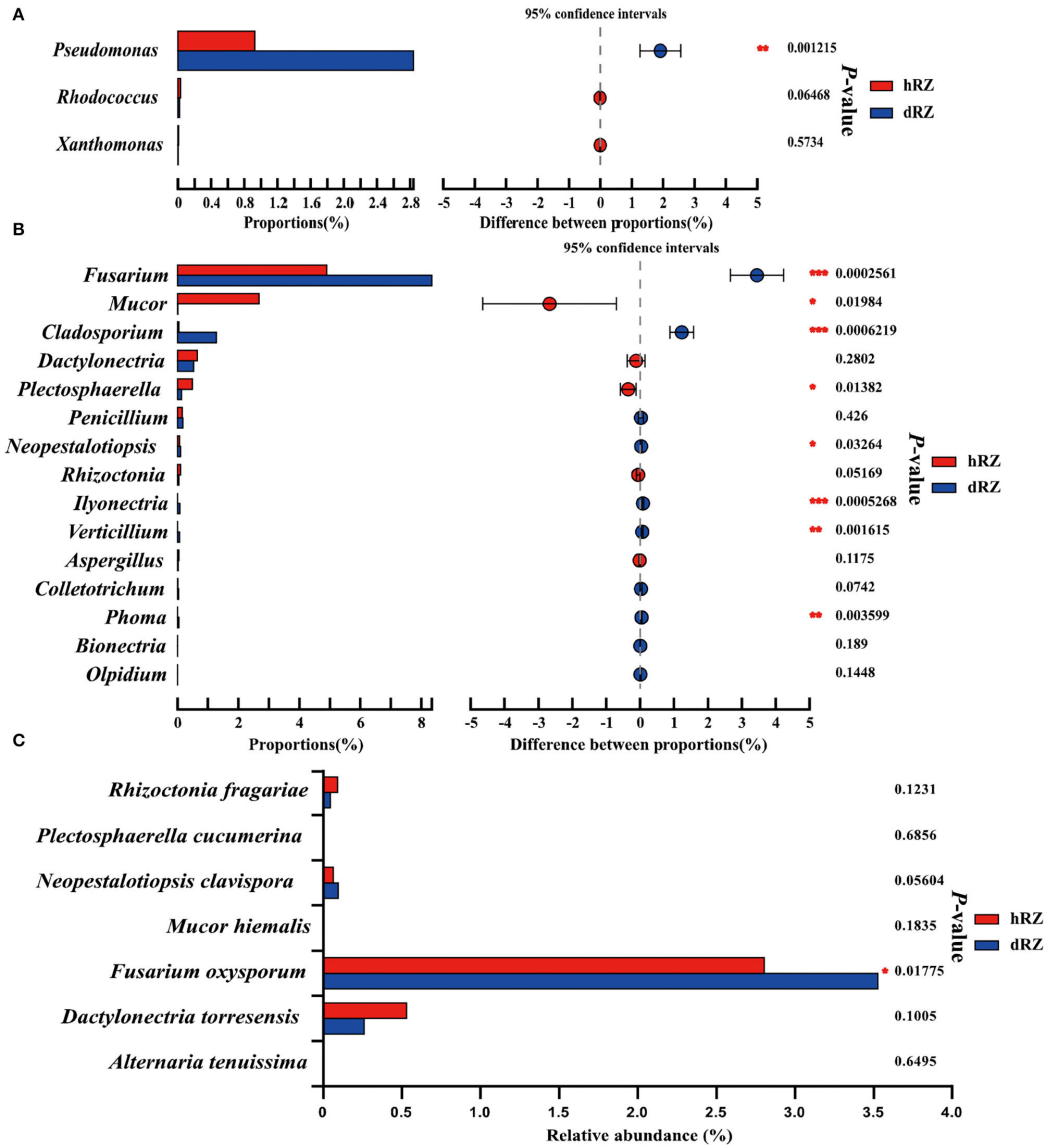
Relative abundances of bacterial **(A)** and fungal **(B)** pathogens between healthy and diseased rhizosphere soils at the genus level and **(C)** fungal pathogen species between healthy and diseased rhizosphere soils. *Indicates significantly different values, *0.01 < *P* ≤ 0.05, **0.001 < *P* ≤ 0.01, ***P ≤ 0.001. *P*-values are based on Student's *t*-tests. hRZ, healthy rhizosphere soils; dRZ, diseased rhizosphere soils (*n* = 3).

According to the APS, it is reported that bacterial pathogens, including *Xanthomonas fragariae, Pseudomonas solanacearum, Rhodococcus fascians* (= *Corynebacterium fascians*), and *Aphelanchoides fragariae*, can cause bacterial diseases in strawberries; more than 50 fungal pathogens can cause fungal diseases in strawberries (https://www.apsnet.org/edcenter/resources/commonnames/Pages/Strawberry.aspx). In this study, the bacterial pathogens were not annotated at the species level (data not shown); the relative abundances of genera *Pseudomonas, Rhodococcus*, and *Xanthomonas* in hRZ and dRZ were different; and the relative abundance of *Pseudomonas* in dRZ was significantly higher than in hRZ, while the relative abundances of *Rhodococcus* and *Xanthomonas* in hRZ were higher than in dRZ (Figure 5A). At the genus level, 15 potential fungal pathogen genera were identified and eight genera were significantly different between healthy and diseased rhizosphere soils (Figure 5B). Furthermore, seven fungal pathogens species, including *Rhizoctonia fragariae, Plectosphaerella cucumerina, Neopestalotiopsis clavispora, Mucor hiemalis, Fusarium oxysporum, Dactylonectria torresensis*, and *Alternaria tenuissima*, were annotated at the species level in two soil groups in this study, and the relative abundance of *F. oxysporum* was significantly different between hRZ and dRZ (Figure 5C).

**Figure 6:**
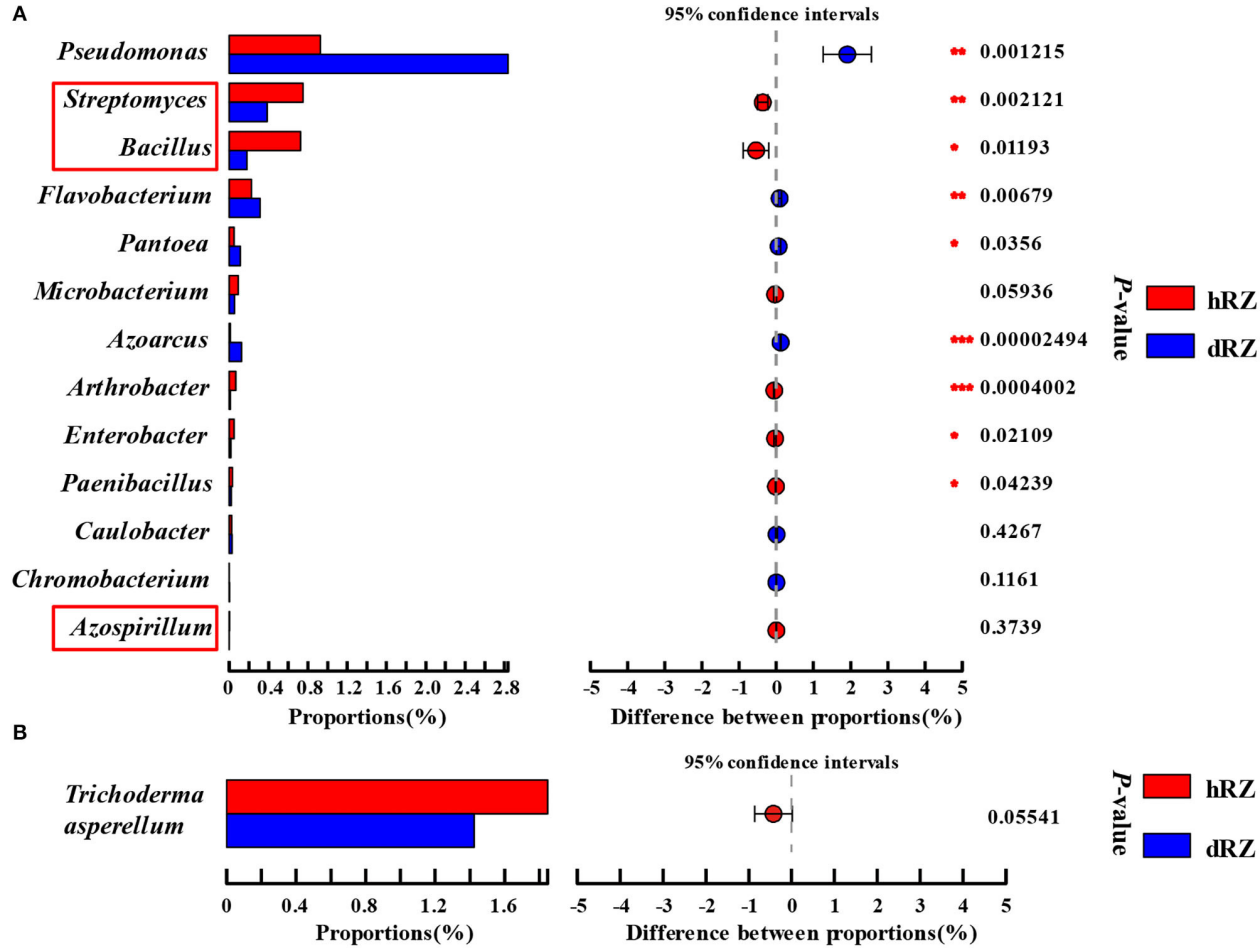
Relative abundances of beneficial bacterial genera **(A)** and fungal species *Trichoderma asperellum* **(B)** both in healthy and diseased rhizosphere soils. *Indicates significantly different values, *0.01 < *P* ≤ 0.05, **0.001 < *P* ≤ 0.01, ***P ≤ 0.001. *P*-values are based on Student's *t*-tests. The red box indicates the antagonistic bacteria species used in strawberries. hRZ, healthy rhizosphere soils; dRZ, diseased rhizosphere soils (*n* = 3).

As reported by some researchers, bacterial species within *Streptomyces* (Marian et al., 2020), *Azospirillum* (Tortora et al., 2011), and *Bacillus* (Mochizuki et al., 2012) can be used to biocontrol strawberry anthracnose. The relative abundances of *Streptomyces* and *Bacillus* in healthy rhizosphere soils were significantly higher than in diseased rhizosphere soils, and *Azospirillum* was only detected in healthy rhizosphere soils (Figure 6A). *Bacillus, Paenibacillus, Streptomyces, Pseudomonas, Flavobacterium, Arthrobacter, Microbacterium, Azoarcus, Azospirillum, Caulobacter, Chromobacterium, Enterobacter*, and *Pantoea* were usually seen as the source of beneficial microbes (Etcheverry et al., 2009; Ahemad and Kibret, 2014; Bruto et al., 2014; Jha and Saraf, 2015; He et al., 2020). In this study, the relative abundances of *Azoarcus, Pantoea, Flavobacterium*, and *Pseudomonas* were significantly increased when the plant was infected, and the relative abundances of *Chromobacterium* and *Caulobacter* also increased, but not significant; the relative abundances of *Paenibacillus, Enterobacter*, and *Arthrobacter* were significantly decreased after strawberry plant was diseased (*Microbacterium* excepted) (Figure 6A). Zhao et al. (2020) and Karimi et al. (2017) reported that *Trichoderma asperellum* can be used as a biocontrol fungus to control strawberry anthracnose caused by *C. fragariae* and *C. nymphaeae*, respectively. In this study, *T. asperellum* was detected between healthy and diseased samples in rhizosphere soils, and the relative abundance in hRZ was higher than in dRZ (Figure 6B).

### Potential Functions of Healthy and Diseased Rhizosphere Soils

In this study, PICRUSt2 (Douglas et al., 2020) and FunGuild (Nguyen et al., 2016) programs were used to predict the potential functions of the prokaryotic and fungal communities within healthy and diseased rhizosphere soils, respectively. For prokaryotic communities, six level 1 KO groups containing 45 level 2 KO groups [metabolism (12/45), genetic information processing (4/45), organismal systems (10/45), cellular processes (4/45), human diseases (12/45), and environmental information processing (3/45)] were identified (Supplementary Tables 2, 3); two of the 45 level 2 KO groups showed different abundances (*P* <0.05) between the two soil groups (Supplementary Table 3 marked red). For fungal communities, the OTUs were annotated into 10 trophic modes, and four modes showed significant differences between healthy and diseased rhizosphere soils (Supplementary Table 4); 20 guilds were annotated, and six guilds showed significant differences between two soil groups (Supplementary Table 5 marked red).

## Discussion

In this study, the high-throughput sequencing technology was used to explore the rhizosphere soil microbial diversity and community structure between healthy and anthracnose-infected strawberry plants in an open field. The study provides new insight into the rhizosphere soil community differences in healthy and anthracnose-infected strawberry plants.

Like animal gut microbes, microbes growing in association with plant roots (in the rhizosphere) can affect the health of their host (Haney et al., 2015). The unbalance of microbial community will lead to plant disease, and the microbial community structure will also change after the plant was infected with pathogens (Hamel et al., 2005). Feng et al. (2018) study revealed that after *Torreya grandis* was infected by the *F. oxysporum* species complex, the growth and vitality of *T. grandis*, soil organic carbon content, and soil microbial abundance were decreased. Lee et al. (2021) investigated that the relative abundances of Actinobacteria and Firmicutes phyla were lower in diseased rhizosphere soil than in healthy rhizosphere soil, and disruption of two phyla caused the incidence of bacterial wilt disease. Chen et al. (2020) studied the endophytic phyllosphere microbiota between healthy and *Pseudomonas syringae* pv. tomato (*Pst*) DC3000-infected *Arabidopsis* quadruple mutants, and the results showed that the unbalance of relative abundance between Proteobacteria and Firmicutes was the main cause of the disease. In this study, the relative abundances of Actinobacteria and Firmicutes were significantly decreased in diseased rhizosphere soils compared with healthy samples, and this may be the main reason that strawberry plant was diseased.

It has been suggested that lower microbial diversity in the rhizosphere soils may lead to a higher occurrence of soil-borne disease (Bailey and Lazarovits, 2003), and most studies revealed that the rhizosphere soil microbial diversity in diseased samples was lower than that in healthy samples (Gorissen et al., 2004; Qiu et al., 2012; Deng et al., 2015; Lai et al., 2019; Yang et al., 2020a). Xiao et al. (2017) used high-throughput sequencing to comparatively study the rhizosphere soil bacterial communities of healthy strawberry and anthracnose-infected strawberry plants, and their results revealed that both OTU numbers and diversity index in infected rhizosphere soil were reduced when compared with the healthy samples. Other researchers' studies revealed that after crops, such as cotton (Liu et al., 2019), potato (Rosenzweig et al., 2012; Zhao et al., 2021), chili (Lai et al., 2019), *Panax notoginseng* (Wu et al., 2015), tobacco (Xiang et al., 2019), and monkshood (Li et al., 2021b), were diseased, the microbial diversity was decreased when compared with healthy samples. However, other researchers also found that when the microbial structure changes, plant roots may be more vulnerable to pathogen infection (Wu et al., 2016), and the microbial diversity in infected samples was higher than in healthy samples. Yang et al. (2021) studied the rhizosphere soil bacterial structures between healthy and root rot apples and found that the bacterial diversity in the rhizosphere soil of root rot apple plant was increased, and the balance of Actinobacteria and Acidobacteria might play an important role in keeping the health of rhizosphere soil. The study on the microbial differences between healthy and diseased crops, such as *P. notoginseng* (Wu et al., 2016), cherry trees (Ding et al., 2021), and banana (Fan et al., 2021), also revealed that diseased samples had higher microbial diversity than healthy samples. In this study, the results showed that both prokaryotic and fungal alpha diversities and OTU numbers in anthracnose-infected strawberry rhizosphere soils were higher than those of healthy strawberry rhizosphere soils, and our results coincide with other studies.

Rhizosphere microorganisms promote plant growth and protect plants from pathogen attack by a range of mechanisms, such as biofertilization, stimulation of root growth, rhizoremediation, control of abiotic stress, and disease control (Mendes et al., 2013). In this study, potentially beneficial bacteria in healthy strawberry rhizosphere soils were much more than in anthracnose-infected plants, and isolation of antagonistic bacteria from healthy samples may be much easier than from diseased samples. Species within *Trichoderma* were widely studied for biocontrol of strawberry fungal diseases, such as black root rot (Asad-Uz-Zaman et al., 2015), *Armillaria* root rot (Raziq and Fox, 1999, 2005; Pellegrini et al., 2014), root rot (El-Fiki and Ahmed, 2017), powdery mildew (Fiamingo et al., 2007), and strawberry anthracnose (Freeman et al., 2004; Porras et al., 2009; Karimi et al., 2017; Zhao et al., 2020). In this study, the relative abundances of *Trichoderma* in hRZ (1.881%) and dRZ (1.452%) were similar and did not show a significant difference (*P* > 0.05) (data not shown), but the beneficial fungal species *T. asperellum* showed a higher relative abundance in healthy strawberry rhizosphere soils. Wei et al. (2010) isolated a fungal strain MT-06 from anthracnose-infected strawberry tissues, and the strain showed an activity for biocontrol of strawberry anthracnose. In this study, species *T. asperellum* was identified in both healthy and diseased strawberry rhizosphere soils, that is, antagonists may be isolated not only from healthy samples but also from diseased samples.

For the sustainable development of the strawberry planting industry, disease biocontrol will be the main research direction in future. Our current research has laid a preliminary foundation for the isolation and identification of strawberry antagonists. Previous studies showed that species within *Agrobacterium, Arthrobacter, Azoarcus, Azotobacter, Azospirillum, Bacillus, Burkholderia, Caulobacter, Chromobacterium, Enterobacter, Erwinia, Flavobacterium, Klebsiella, Micrococcous, Rhizobium, Pantoea, Pseudomonas, Microbacterium, Serratia, Paenibacillus*, and *Streptomyces* had the potential roles in promoting plant growth and inhibiting different fungal pathogens (Etcheverry et al., 2009; Ahemad and Kibret, 2014; Bruto et al., 2014; Jha and Saraf, 2015; He et al., 2020). Patel et al. (2021) used the cultured method to select growth promotion isolates from peanut rhizosphere soil, and *Burkholderia* sp. RR18 was the most effective isolate; RR18 had activities, such as reduced collar rot disease incidence, increased germination rate and biomass of peanut seeds, and increased broad-spectrum antifungal activity. Li et al. (2021c) study revealed that after *Astragalus mongholicus* root infected by *F. oxysporum*, the plant roots can recruit some beneficial microbes, including *Pseudomonas, Strenotrophomonas, Chryseobacterium, Achromobacter*, and *Flavobacterium*. Wu et al. (2021) investigated the differences between *Chinese wheat yellow mosaic* resistant and susceptible wheat root endosphere and rhizosphere microbial, and the results revealed that beneficial rhizosphere microbes, such as *Xanthomonadales, Actinomycetales, Sphingomonas, Rhizobium, Bacillaceae, Bacillus, Streptomycetaceae, Streptomyces, Nocardioides, Pseudonocardia, Bradyrhizobium, Pseudonocardiaceae*, and *Solibacteraceae*, were enriched in the resistant wheat root. Some studies revealed that potential beneficial microbes, such as *Bacillus, Paenibacillus, Streptomyces, Pseudomonas, Flavobacterium, Arthrobacter, Rhizobium, Microbacterium, Micrococcous*, and *Burkholderia*, can establish disease suppression by a single strain or SynComs (synthetic microbial communities) *via* an antagonistic effect to protect the host from pathogens' invasion and attack (Weller et al., 2002; Lee et al., 2021; Li et al., 2021c; Marín et al., 2021). In this study, *Streptomyces, Azospirillum*, and *Bacillus* were identified as the potentially beneficial bacterial genera; some studies show that species within these genera can be used to biocontrol strawberry anthracnose; other beneficial microbes, such as *Chromobacterium, Caulobacter, Paenibacillus, Enterobacter, Arthrobacter, Azoarcus, Microbacterium, Pantoea, Flavobacterium*, and *Pseudomonas*, were also detected in rhizosphere soils, and their relative abundances were changed after plants were infected by pathogens. We speculated that when strawberry plants were invaded by pathogens, on the one hand, the rhizosphere soil microbial community was unbalanced and showed symptoms, on the other hand, plants will recruit probiotic groups to resist the further expansion of pathogens, so the relative abundances of beneficial microbes, such as *Chromobacterium, Caulobacter, Azoarcus, Pantoea, Flavobacterium*, and *Pseudomonas*, were higher in diseased samples than in healthy samples, and meanwhile, species within these groups may be the main source of probiotic bacteria. However, to determine whether these beneficial microbes can protect strawberry plants from pathogens infection, they need to be further isolated and tested in the laboratory.

When we collected strawberry rhizosphere soil samples, angular leaf spot-like symptoms were observed on plants from this strawberry-planted field. The angular leaf spot pathogen *X. fragariae* was not identified in this study, and the relative abundance of *Xanthomonas* was similar in the two soil samples; other bacterial pathogens, such as *P. solanacearum, R. fascians*, and *A. fragariae*, were still not identified. Some studies revealed that species within *Colletotrichum* could not only cause strawberry anthracnose but also cause other fungal diseases, such as crown and root rot (Han et al., 2014; Zhang et al., 2015). Referring to the APS, eight of 15 potential fungal pathogen genera were identified and showed significant differences between healthy and diseased strawberry rhizosphere soils in this study. Among seven potential fungal pathogen species, the relative abundance of *F. oxysporum* was significantly higher in diseased strawberry rhizosphere soils. The sampling field has planted strawberries for 4 years, soils maybe contained spores of pathogenic fungi, and the fungal pathogen species were identified in this study. Based on the above research, we can speculate that strawberries planted in this area were at risk of other diseases besides strawberry anthracnose.

## Conclusion

In this study, Illumina sequencing was used to comparatively study the rhizosphere soil microbial communities between healthy and anthracnose-infected strawberry plants. Both rhizosphere soil community diversities and richness of the anthracnose-infected strawberry rhizosphere soils were higher than those of the healthy plants. When strawberry plants were infected, the diversities and compositions of rhizosphere soil microbial community were changed. Based on this, the potential probiotics and pathogens in the samples were comparatively analyzed, the potential probiotic abundances in healthy samples were higher than in diseased samples, and the changes of pathogens did not show a regularity. The results of this article will help researchers to carry out culture-dependent work and provide a basis for the isolation and identification of strawberry pathogens and probiotics.

## Data Availability Statement

Raw sequence data reported in this paper has been deposited to the National Center for Biotechnology Information (NCBI) under accession number PRJNA802716 (16S rRNA) and PRJNA802988 (ITS).

## Author Contributions

DS, SC, JY, JT, and XC planned and designed the research and performed the experiments. DS, SC, WZ, JY, ZL, ZZ, YT, QD, XS, and SW performed the experiments. DS, SC, WZ, and JY analyzed the data. DS, JT, and XC wrote the manuscript. SW, JT, and XC acquired the funds for the study. All authors read and improved the manuscript and agreed to the publication of the final manuscript.

## Funding

This work was supported by grants from the National Natural Science Foundation of China (Grant Nos. 31960220 and 31660089).

## Conflict of Interest

The authors declare that the research was conducted in the absence of any commercial or financial relationships that could be construed as a potential conflict of interest.

## Publisher's Note

All claims expressed in this article are solely those of the authors and do not necessarily represent those of their affiliated organizations, or those of the publisher, the editors and the reviewers. Any product that may be evaluated in this article, or claim that may be made by its manufacturer, is not guaranteed or endorsed by the publisher.

## References

[B1] Abarenkov K. Nilsson R. H. Larsson K. H. Alexander I. J. Eberhardt U. Erland S.. (2010). The UNITE database for molecular identification of fungi - recent updates and future perspectives. New Phytol. 186, 281–285. 10.1111/j.1469-8137.2009.03160.x 20409185

[B2] Adhikari T. B. Chacon J. G. Fernandez G. E. Louws G. J. (2019). First report of anthracnose causing both crown and fruit rot of strawberry by *Colletotrichum siamense* in North Carolina. Plant Dis. 103, 1775–1775. 10.1094/PDIS-02-19-0314-PDN

[B3] Ahemad M. Kibret M. (2014). Mechanisms and applications of plant growth promoting rhizobacteria: current perspective. J. King Saud Univ. Sci. 26, 1–20. 10.1016/j.jksus.2013.05.001

[B4] Amann R. I. Ludwig W. Schleifer K. H. (1995). Phylogenetic identification of individual microbial cells without cultivation. Microbiol. Rev. 59, 143–169. 10.1128/mr.59.1.143-169.1995 7535888 PMC239358

[B5] Asad-Uz-Zaman M. Bhuiyan M. R. Khan M. A. I. Bhuiyan M. K. I. Latif M. A. (2015). Integrated options for the management of black root rot of strawberry caused by *Rhizoctonia solani* Kuhn. C. R. Biol. 338, 112–120. 10.1016/j.crvi.2014.11.006 25595298

[B6] Bailey K. L. Lazarovits G. (2003). Suppressing soil-borne diseases with residue management and organic amendments. Soil Tillage Res. 72, 169–180. 10.1016/S0167-1987(03)00086-2

[B7] Bi Y. Guo W. Zhang G. J. Liu S. C. Chen Y. (2017). First report of *Colletotrichum truncatum* causing anthracnose of strawberry in China. Plant Dis. 101, 832–832. 10.1094/PDIS-07-16-1036-PDN 30708530

[B8] Bolger A. M. Lohse M. Usadel B. (2014). Trimmomatic: a flexible trimmer for Illumina sequence data. Bioinf. 30, 2114–2120. 10.1093/bioinformatics/btu170 24695404 PMC4103590

[B9] Bowya T. Balachandar D. (2020). Rhizosphere engineering through exogenous growth-regulating small molecules improves the colonizing efficiency of a plant growth-promoting rhizobacterium in rice. 3 Biotech 10:277. 10.1007/s13205-020-02275-5 32537377 PMC7261316

[B10] Brooks A. N. (1931). Anthracnose of strawberry caused by *Colletotrichum fragariae*. Phytopathol. 21, 739–744.34603266

[B11] Bruto M. Prigent-Combaret C. Muller D. Moënne-Loccoz Y. (2014). Analysis of genes contributing to plant-beneficial functions in plant growth-promoting rhizobacteria and related Proteobacteria. Sci. Rep. 4:6261. 10.1038/srep06261 25179219 PMC4151105

[B12] Chen T. Nomura K. Wang X. L. Sohrabi R. Xu J. Xu L. Y.. (2020). A plant genetic network for preventing dysbiosis in the phyllosphere. Nature 580, 653–657. 10.1038/s41586-020-2185-0 32350464 PMC7197412

[B13] Cheng X. F. Zhang M. Adhikari B. (2014). Effect of ultrasonically induced nucleation on the drying kinetics and physical properties of freeze-dried strawberry. Drying Technol. 32, 1857–1864. 10.1080/07373937.2014.952741

[B14] Choi K. H. Dobbs F. C. (1999). Comparison of two kinds of biolog microplates (GN and ECO) in their ability to distinguish among aquatic microbial communities. J. Microbiol. Methods 36, 203–213. 10.1016/S0167-7012(99)00034-2 10379806

[B15] Coleman-Derr D. Desgarennes D. Fonseca-Garcia C. Gorss S. Clingenpeel S. Woyke T.. (2016). Plant compartment and biogeography affect microbiome composition in cultivated and native *Agave* species. New Phytol. 209, 798–811. 10.1111/nph.13697 26467257 PMC5057366

[B16] Cui Y. X. Bing H. J. Fang L. C. Wu Y. H. Yu J. L. Shen G. T.. (2019). Diversity patterns of the rhizosphere and bulk soil microbial communities along an altitudinal gradient in an alpine ecosystem of the eastern Tibetan Plateau. Geoderma 338, 118–127. 10.1016/j.geoderma.2018.11.047

[B17] Dai F. M. Ren X. J. Lu J. P. (2006). First report of anthracnose fruit rot of strawberry caused by *Colletotrichum acutatum* in China. Plant Dis. 90, 1460–1460. 10.1094/PD-90-1460A 30780923

[B18] Dai T. Chang X. N. Hu Z. H. Liang L. Sun M. Y. Liu P. F.. (2019). Untargeted metabolomics based on GC-MS and chemometrics: a new tool for the early diagnosis of strawberry anthracnose caused by *Colletotrichum theobromicola*. Plant Dis. 103, 2541–2547. 10.1094/PDIS-01-19-0219-RE 31432772

[B19] Deng X. Li Q. F. Wu C. Y. Li Y. Liu J. K. (2015). Comparison of soil bacterial genetic diversity in root zone of banana (*Musa paradisiaca*) infected with *Fusarium wilt* and non-infected plants. Ecol. Environ. Sci. 24:402–408. 10.16258/j.cnki.1674-5906.2015.03.006

[B20] Denoyes-Rothan B. Guérin G. Délye C. Smith B. Minz D. Maymon M.. (2003). Genetic diversity and pathogenic variability among isolates of *Colletotrichum* species from strawberry. Phytopathology. 93, 219–228. 10.1094/PHYTO.2003.93.2.219 18943137

[B21] Desgarennes D. Garrido E. Torres-Gomez M. J. Peña-Cabriales J. J. Partida-Martinez L. P. (2014). Diazotrophic potential among bacterial communities associated with wild and cultivated *Agave* species. FEMS Microbiol. Ecol. 90, 844–857. 10.1111/1574-6941.12438 25314594

[B22] Ding L. Xu L. J. Chu X. Yang L. Zhu H. L. Huang J. X. (2021). Dissimilarity analysis of microbial communities in the rhizosphere and tissues of diseased and healthy cherry trees (*Cerasus pseudocerasus*). Can. J. Plant Pathol. 43, 612–621. 10.1080/07060661.2020.1861101

[B23] Douglas G. M. Maffei V. J. Zaneveld J. R. Yurgel S. N. Brown J. R. Taylor C. M.. (2020). PICRUSt2 for prediction of metagenome functions. Nat. Biotechnol. 38, 685–688. 10.1038/s41587-020-0548-6 32483366 PMC7365738

[B24] Edgar R. C. (2013). UPARSE: highly accurate OTU sequences from microbial amplicon reads. Nat. Methods 10, 996–998. 10.1038/nmeth.2604 23955772

[B25] Edwards J. Johnson C. Santos-Medellín C. Lurie E. Podishetty N. K. Bhatnagar S.. (2015). Structure, variation, and assembly of the root-associated microbiomes of rice. Proc. Natl. Acad. Sci. USA 112, E911–E920. 10.1073/pnas.1414592112 25605935 PMC4345613

[B26] El-Fiki I. A. I. Ahmed M. F. A. (2017). Effect of biological control of root rot diseases of strawberry using *Trichoderma* spp. Res. J. Appl. Sci. 7, 482–492.30780995

[B27] Etcheverry M. G. Scandolara A. Nesci A. Ribeiro M. S. V. B. Pereira P. Battilani P. (2009). Biological interactions to select biocontrol agents against toxigenic strains of *Aspergillus flavus* and *Fusarium verticillioides* from Maize. Mycopathologia 167, 287–295. 10.1007/s11046-008-9177-1 19247799

[B28] Fan H. C. Wei W. Zeng L. Xu S. T. Li S. Guo Z. X.. (2021). Comparative analysis on difference of bacterial community structure in rhizosphere soil between banana *Fusarium* wilt and healthy plants. Southwest China J. Agric. Sci. 24, 1885–1891. 10.16213/j.cnki.scjas.2021.9.011

[B29] Feng Y. X. Hu Y. Y. Wu J. S. Chen J. H. Yrjälä K. Yu W. W. (2018). Change in microbial communities, soil enzyme and metabolic activity in a *Torreya grandis* plantation in response to root rot disease. For. Ecol. Manage. 432, 932–941. 10.1016/j.foreco.2018.10.028

[B30] Fiamingo F. Elad Y. Pertot I. (2007). Effect of application time of control agents on *Podosphaera aphanis* and side effect of fungicides on biocontrol agents survival on strawberry leaves. pp. 433–436.

[B31] Freeman S. Katan T. (1997). Identification of *Colletotrichum* species responsible for anthracnose and root necrosis of strawberry in Israel. Phytopathol. 87, 516–521. 10.1094/PHYTO.1997.87.5.516 18945106

[B32] Freeman S. Minz D. Kolesnik I. Barbul O. Zveibil A. Maymon M.. (2004). *Trichoderma* biocontrol of *Colletotrichum acutatum* and *Botrytis cinerea* and survival in strawberry. Eur. J. Plant Pathol. 110, 361–370. 10.1023/B:EJPP.0000021057.93305.d9

[B33] Garrido C. Carbú M. Fernández-Acero F. J. Budge G. E. Vallejo I. Colyer A.. (2008). Isolation and pathogenicity of *Colletotrichum* spp. causing anthracnose of strawberry in south west Spain. Eur. J. Plant Pathol. 120, 409–415. 10.1007/s10658-007-9224-7

[B34] Garrido C. Carbú M. Fernández-Acero F. J. González-Rodríguez V. E. Cantoral J. M. (2011). New insights in the study of strawberry fungal pathogens. Genes Genomes Genomics 5, 24–39.

[B35] Gorissen A. van Overbeek L. S. van Elsas J. D. (2004). Pig slurry reduces the survival of *Ralstonia solanacearum* biovar 2 in soil. Can. J. Microbiol. 50, 587–593. 10.1139/w04-042 15467784

[B36] Hamel C. Vujanovic V. Jeannotte R. Nakano-Hylander A. St-Arnaud M. (2005). Negative feedback on a perennial crop: *Fusarium* crown and root rot of asparagus is related to changes in soil microbial community structure. Plant Soil 268, 75–87. 10.1007/s11104-004-0228-1

[B37] Han Y. C. Xiang F. Y. Zeng X. G. Zhang P. Gu Y. C. (2014). Identification of pathogen causing crown and root rot on strawberry. Sci. Agric. Sin. 47, 53–60. 10.3864/j.issn.0578-1752.2014.01.00630722455

[B38] Haney C. H. Samuel B. S. Bush J. Ausubel F. M. (2015). Associations with rhizosphere bacteria can confer an adaptive advantage to plants. Nat. Plants 1:15051. 10.1038/nplants.2015.51 27019743 PMC4806546

[B39] He Z. D. Gao Y. F. Wang Y. Li C. X. Gao X. Y. Zhang Z. H. (2020). Study on phosphate-solubilizing strain selection of plant growth promoting rhizobacteria and its effect on tomato growth promotion. Southwest China J. Agric. Sci. 33, 2891–2896. 10.16213/j.cnki.scjas.2020.12.030

[B40] Hoseini S. Amini J. Nazemi-Rafie J. Khorshidi J. (2019). Inhibitory effect of some plant essential oils against strawberry anthracnose caused by *Colletotrichum nymphaeae* under *in vitro* and *in vivo* conditions. Eur. J. Plant Pathol. 155, 1287–1302. 10.1007/s10658-019-01856-2

[B41] Hu M. (1990). The preliminary investigation of strawberry diseases, pp. 58–59, Jiangsu.

[B42] Husaini A. M. Neri D. (2016). Strawberry Growth, Development and Diseases. CAB International. 10.1079/9781780646633.0000

[B43] Jayawardena R. S. Huang J. K. Jin B. C. Yan J. Y. Li X. H. Hyde K. D.. (2016). An account of *Colletotrichum* species associated with strawberry anthracnose in China based on morphology and molecular data. Mycosphere 7, 1147–1163. 10.5943/mycosphere/si/2c/6

[B44] Jha C. K. Saraf M. (2015). Plant growth promoting rhizobacteria (PGPR): a review. E3 J. Agric. Res. Dev. 5, 108–119. 10.13140/RG.2.1.5171.216433808829

[B45] Karimi K. Ahari A. B. Arzanlou M. Amini J. Pertot I. (2017). Comparison of indigenous Trichoderma spp. strains to a foreign commercial strain in terms of biocontrol efficacy against *Colletotrichum nymphaeae* and related biological features. J. Plant Dis. Prot. 124, 453–466. 10.1007/s41348-017-0088-6

[B46] Karimi K. Babai-Ahari A. Arzanlou M. (2015). Strawberry anthracnose disease. Plant Pathol. Sci. 4, 26–40.

[B47] Lai B. C. Dai R. Q. Wu Z. Q. Li F. Lin D. F. Wang J. R. (2019). Bacterial diversities in rhizosphere soils at sites of healthy and *Fusarium* wilt infected chili plants. Fujian J. Agric. Sci. 34, 1073–1080. 10.19303/j.issn.1008-0384.2019.09.012

[B48] Lee S. M. Kong H. G. Song G. C. Ryu C. M. (2021). Disruption of Firmicutes and Actinobacteria abundance in tomato rhizosphere causes the incidence of bacterial wilt disease. ISME J. 15, 330–347. 10.1038/s41396-020-00785-x 33028974 PMC7852523

[B49] Li J. Y. Zhao Q. Q. Wuriyanghan H. D. Yang C. (2021a). Biocontrol bacteria strains Y4 and Y8 alleviate tobacco bacterial wilt disease by altering their rhizosphere soil bacteria community. Rhizosphere 19:100390. 10.1016/j.rhisph.2021.100390

[B50] Li X. G. Panke-Buisse K. Yao X. D. Coleman-Derr D. Ding C. F. Wang X. X.. (2020). Peanut plant growth was altered by monocropping-associated microbial enrichment of rhizosphere microbiome. Plant Soil 446, 655–669. 10.1007/s11104-019-04379-1

[B51] Li Y. L. He F. Guo Q. Feng Z. Y. Zhang M. Ji C. L.. (2021b). Compositional and functional comparison on the rhizosphere microbial community between healthy and Sclerotium rolfsii-infected monkshood (*Aconitum carmichaelii*) revealed the biocontrol potential of healthy monkshood rhizosphere microorganisms. Biol. Control 165:104790. 10.1016/j.biocontrol.2021.104790

[B52] Li Z. F. Bai X. L. Jiao S. Li Y. M. Li P. R. Yang Y.. (2021c). A simplified synthetic community rescues *Astragalus mongholicus* from root rot disease by activating plant-induced systemic resistance. Microbiome 9:217. 10.1186/s40168-021-01169-9 34732249 PMC8567675

[B53] Liao C. Y. Chen M. Y. Chen Y. K. Wang T. C. Sheu Z. M. Kuo K. C.. (2012). Characterization of three *Colletotrichum acutatum* isolates from *Capsicum* spp. Eur. J. Plant Pathol. 133, 599–608. 10.1007/s10658-011-9935-7

[B54] Liu H. Y. Wang W. Zhang R. F. Abdurahman R. Yao J. (2019). Fungal community structure of cotton-field soil under different incidences of cotton *Verticillium* wilt. Sci. Agric. Sin. 52, 455–465. 10.3864/j.issn.0578-1752.2019.03.006

[B55] Liu Y. Ji Y. Han Y. C. Song L. L. Zhang L. Q. Ning Z. Y.. (2021). Loop-mediated isothermal amplification and PCR combined assay to detect and distinguish latent *Colletotrichum* spp. infection on strawberry. J. Plant Pathol. 103, 887–99. 10.1007/s42161-021-00873-7

[B56] Lu X. Chen L. H. Li Y. R. (2012). Effect of DNA extraction on DGGE analysis of microbial community in soil. Fujian J. Agric. Sci. 27, 367–372. 10.19303/j.issn.1008-0384.2012.04.011

[B57] Magoč T. Salzberg S. L. (2011). FLASH: fast length adjustment of short reads to improve genome assemblies. Bioinformatics. 27, 2957–2963. 10.1093/bioinformatics/btr507 21903629 PMC3198573

[B58] Marian M. Ohno T. Suzuki H. Kitamura H. Kuroda K. Shimizu M. (2020). A novel strain of endophytic *Streptomyces* for the biocontrol of strawberry anthracnose caused by *Glomerella cingulate*. Microbiol. Res. 234:126428. 10.1016/j.micres.2020.126428 32086186

[B59] Marín O. González B. Poupin M. J. (2021). From microbial dynamics to functionality in the rhizosphere: a systematic review of the opportunities with synthetic microbial communities. Front. Plant Sci. 12:650609. 10.3389/fpls.2021.650609 34149752 PMC8210828

[B60] Mass J. L. (1978). Anthracnose of strawberry fruit in Maryland. Plant Dis. 62, 488–492.

[B61] Mendes R. Garbeva P. Raaijmakers J. M. (2013). The rhizosphere microbiome: significance of plant beneficial, plant pathogenic, and human pathogenic microorganisms. FEMS Microbiol. Rev. 37, 634–663. 10.1111/1574-6976.12028 23790204

[B62] Mertely J. C. Legard D. E. (2004). Detection, isolation, and pathogenicity of *Colletotrichum* spp. from strawberry petioles. Plant Dis. 88, 407–418. 10.1094/PDIS.2004.88.4.407 30812623

[B63] Mochizuki M. Yamamoto S. Aoki Y. Suzuki S. (2012). Isolation and characterisation of *Bacillus amyloliquefaciens* S13-3 as a biological control agent for anthracnose caused by *Colletotrichum gloeosporioides*. Biocontrol Sci. Technol. 22, 697–709. 10.1080/09583157.2012.679644

[B64] Nguyen N. H. Song Z. W. Bates S. T. Branco S. Tedersoo L. Menke J.. (2016). FUNGuild: an open annotation tool for parsing fungal community datasets by ecological guild. Fungal Ecol. 20, 241–248. 10.1016/j.funeco.2015.06.006

[B65] Pantigoso H. A. Manter D. K. Vivanco J. M. (2020). Differential effects of phosphorus fertilization on plant uptake and rhizosphere microbiome of cultivated and non-cultivated potatoes. Microb. Ecol. 80, 169–180. 10.1007/s00248-020-01486-w 32016609

[B66] Patel R. R. Patel D. D. Bhatt J. Thakor P. Triplett L. R. Thakkar V. R. (2021). Induction of pre-chorismate, jasmonate, and salicylate pathways by *Burkholderia* sp. RR18 in peanut seedlings. J. Appl. Microbiol. 131, 1417–1430. 10.1111/jam.15019 33522007

[B67] Pellegrini A. Prodorutti D. Pertot I. (2014). Use of bark mulch pre-inoculated with *Trichoderma atroviride* to control *Armillaria* root rot. Crop Prot. 64, 104–109. 10.1016/j.cropro.2014.06.007

[B68] Peres N. A. Timmer L. W. Adaskaveg J. E. Correll J. C. (2005). Lifestyles of *Colletotrichum acutatum*. Plant Dis. 89, 784–796. 10.1094/PD-89-0784 30786507

[B69] Pérez-Jaramillo J. E. Mendes R. Raaijmakers J. M. (2016). Impact of plant domestication on rhizosphere microbiome assembly and functions. Plant Mol. Biol. 90, 635–644. 10.1007/s11103-015-0337-7 26085172 PMC4819786

[B70] Phoulivong S. Cai L. Chen H. McKenzie E. H. C. Abdelsalam K. Chukeatirote E.. (2010). *Colletotrichum gloeosporioides* is not a common pathogen on tropical fruits. Fungal Divers. 44, 33–43. 10.1007/s13225-010-0046-0

[B71] Porras M. Barrau C. Romero F. (2009). Biological control of anthracnose with *Trichoderma* in strawberry fields. Acta Hortic. 842, 351–354. 10.17660/ActaHortic.2009.842.66

[B72] Qiu M. H. Zhang R. F. Xue C. Zhang S. S. Li S. Q. Zhang N.. (2012). Application of bio-organic fertilizer can control *Fusarium wilt* of cucumber plants by regulating microbial community of rhizosphere soil. Biol. Fertil. Soils 48, 807–816. 10.1007/s00374-012-0675-4

[B73] Quast C. Pruesse E. Yilmaz P. Gerken J. Schweer T. Yarza P.. (2013). The SILVA ribosomal RNA gene database project: improved data processing and web-based tools. Nucleic Acids Res. 41, D590–D596. 10.1093/nar/gks1219 23193283 PMC3531112

[B74] Raziq F. Fox R. T. V. (1999). Biological control of *Armillaria* root rot. Acta Hortic. 496, 115–126. 10.17660/ActaHortic.1999.496.14

[B75] Raziq F. Fox R. T. V. (2005). Combinations of fungal antagonists for biological control of *Armillaria* root rot of strawberry plants. Biol. Agric. Hortic. 23, 45–57. 10.1080/01448765.2005.9755307

[B76] Reddy P. P. (2016). Sustainable crop protection under protected cultivation. Springer. 10.1007/978-981-287-952-3

[B77] Roose-Amsaleg C. L. Garnier-Sillam E. Harry M. (2001). Extraction and purification of microbial DNA from soil and sediment samples. Appl. Soil. Ecol. 18, 47–60. 10.1016/S0929-1393(01)00149-421519906

[B78] Rosenzweig N. Tiedje J. M. Quensen J. F. Meng Q. X. Hao J. J. J. (2012). Microbial communities associated with potato common scab-suppressive soil determined by pyrosequencing analyses. Plant Dis. 96, 718–725. 10.1094/PDIS-07-11-0571 30727523

[B79] Singh B. Singh S. K. Agarwal P. C. Rani I. Khetarpal R. K. (2003). *Colletotrichum dematium* causing anthracnose in hybrid strawberry (*Fragaria* × *ananassa*)-a new host record for India. Indian J. Agric. Sci. 73, 238–239.

[B80] Su D. F. Tong J. Y. Yang J. Y. Chen S. Y. Luo Z. W. Shen X. M.. (2018). Advances in research, exploitation and utilization of *Fragaria* spp. germplasm resources in China. J. Yunnan Univ. 40, 1261–1276. 10.7540/j.ynu.20180613

[B81] Sun B. Liu J. Meng B. Bao K. (2020). Structural variability and co-occurrence pattern differentiation in rhizosphere microbiomes of the native invasive plant *Echinochloa caudate* in Momoge National Nature Reserve, China. Wetlands 40, 587–597. 10.1007/s13157-019-01209-z

[B82] Tedersoo L. Anslan S. Bahram M. Põlme S. Riit T. Liiv I.. (2015). Shotgun metagenomes and multiple primer pair-barcode combinations of amplicons reveal biases in metabarcoding analyses of fungi. MycoKeys 10, 1–43. 10.3897/mycokeys.10.4852

[B83] Tortora M. L. Díaz-Ricci J. C. Pedraza R. O. (2011). *Azospirillum brasilense* siderophores with antifungal activity against *Colletotrichum acutatum*. Arch. Microbiol. 193, 275–286. 10.1007/s00203-010-0672-7 21234749

[B84] Wang Y. T. Fu L. N. Ji G. H. Yang J. Wang X. Zhang J. H.. (2019). A study of the microbial community diversity of corn rhizosphere in Yunnan province based on high-throughput sequencing technique. Acta Agric. Univ. Jiangxiensis 41, 491–500. 10.13836/j.jjau.2019058

[B85] Wei C. Y. Mao X. Q. Cai R. Y. Wang Y. L. Wang J. Y. Zhang Z.. (2010). Identification and characterization of the fungal strain MT-06 for biocontrol of strawberry anthracnose. Mycosystema 29, 481–487. 10.13346/j.mycosystema.2010.04.017

[B86] Weller D. M. Raaijmakers J. M. Gardener B. B. M. Thomashow L. S. (2002). Microbial populations responsible for specific soil suppressiveness to plant pathogens. Annu. Rev. Phytopathol. 40, 309–348. 10.1146/annurev.phyto.40.030402.110010 12147763

[B87] Wu C. F. Wang F. Y. Zhang H. Q. Chen G. X. Deng Y. W. Chen J. P.. (2021). Enrichment of beneficial rhizosphere microbes in *Chinese wheat yellow mosaic virus*-resistant cultivars. Appl. Microbiol. Biotechnol. 105, 9371–9383. 10.1007/s00253-021-11666-4 34767052

[B88] Wu Z. X. Hao Z. P. Sun Y. Q. Guo L. P. Huang L. Q. Zeng Y.. (2016). Comparison on the structure and function of the rhizosphere microbial community between healthy and root-rot *Panax notoginseng*. Appl. soil Ecol. 107, 99–107. 10.1016/j.apsoil.2016.05.017

[B89] Wu Z. X. Hao Z. P. Zeng Y. Guo L. P. Huang L. Q. Chen B. D. (2015). Molecular characterization of microbial communities in the rhizosphere soils and roots of diseased and healthy *Panax notoginseng*. Antonie van Leeuwenhoek 108, 1059–1074. 10.1007/s10482-015-0560-x 26296378

[B90] Xiang L. G. Wang H. C. Guo Z. N. Xie H. L. Cai L. T. Yu Z. H. (2019). The influence of black shank disease infection on fungal community structure of rhizosphere soil and stem of tobacco plants. Mycosystema 38, 2099–2111. 10.13346/j.mycosystema.190362

[B91] Xiao R. Cao Q. F. Nie Y. J. Zhang C. F. Deng S. Sun H. F.. (2017). A comparative study on rhizosphere soil bacterial communities of healthy strawberry and infected strawberry with anthracnose by high-throughput sequencing. Chin. Agric. Sci. Bull. 33, 14–20.

[B92] Yang G. Z. Huang W. J. Zheng L. P. He Y. Y. Zhang Y. Kong B. H.. (2021). Bacterial community structure and diversity of rhizosphere soil of healthy and root rot apple based on high-throughput sequencing. Southwest China J. Agric. Sci. 34, 1865–1869. 10.16213/j.cnki.scjas.2021.9.008

[B93] Yang J. Y. Wei S. J. Su D. F. Zhang Z. R. Chen S. Y. Luo Z. W.. (2020a). Comparison of the rhizosphere soil microbial community structure and diversity between powdery mildew-infected and noninfected strawberry plants in a greenhouse by high-throughput sequencing technology. Curr. Microbiol. 77, 1724–1736. 10.1007/s00284-020-01948-x 32314037

[B94] Yang J. Y. Wei S. J. Su D. F. Zhang Z. R. Chen S. Y. Luo Z. W.. (2020b). Study on prokaryotic communities in rhizospheres of powdery mildew-infected and non-infected strawberry in greenhouse by high-through sequencing technology. Southwest China J. Agric. Sci. 33, 85–91. 10.16213/j.cnki.scjas.2020.1.014

[B95] Yao X. D. Wang W. Zeng H. (2016). Application of phospholipid fatty acid method in analyzing soil microbial community. Microbiol. China 43, 2086–2095. 10.13344/j.microbiol.china.16001432062778

[B96] Yu Y. Lee C. Kim J. Hwang S. (2010). Group-specific primer and probe sets to detect methanogenic communities using quantitative real-time polymerase chain reaction. Biotechnol. Bioeng. 89, 670–679. 10.1002/bit.20347 15696537

[B97] Zakaria L. (2021). Diversity of *Colletotrichum* species associated with anthracnose disease in tropical fruit crops-a review. Agric. 11:297. 10.3390/agriculture11040297

[B98] Zhang Y. Liu Z. P. Wei Y. M. Shang Q. X. Li Y. Q. Zhao X. Y. (2015). Identification of the strawberry root rot pathogen in Changping District Beijing. Chin. Agric. Sci. Bull. 31, 278–284.

[B99] Zhao D. L. He H. Y. Wu S. P. Chen X. J. Tan Q. Q. Yang X. H. (2020). Biocontrol mechanisms and control effects of *Trichoderma asperellum* GYSW-6m1 on strawberry anthracnose and growth-promoting effects on strawberry. Chin. J. Biol. Control 36, 587–595. 10.16409/j.cnki.2095-039x.2020.04.020

[B100] Zhao F. Zhao M. Z. Wang Y. Guan L. Pang F. H. (2019). Microbial community structures and diversities in strawberry rhizosphere soils based on high-throughput sequencing. Soils 51, 51–60. 10.13758/j.cnki.tr.2019.01.008

[B101] Zhao W. S. Guo Q. G. Su Z. H. Wang P. P. Dong L. H. Hu Q.. (2021). Characterization of fungal community structure in the rhizosphere soil of healthy and diseased-*Verticillium* wilt potato plants and carbon source utilization. Sci. Agric. Sin. 54, 296–309. 10.3864/j.issn.0578-1752.2021.02.006

[B102] Zhou L. T. Li J. J. Zhao Y. L. Luo Y. Bai Y. Chen J.. (2020). Variation of bacterial communities in the rhizosphere soils of successive rotations *Casuarina equisetifolia* plantations based on high-throughput sequencing analysis. Acta. Ecol. Sin. 40, 2670–2679. 10.5846/stxb201902250348

