# Supplementary material for: Comparative Analysis of the Microbial Community Structures Between Healthy and Anthracnose-Infected Strawberry Rhizosphere Soils Using Illumina Sequencing Technology in Yunnan Province, Southwest of China
Source: Front Microbiol. 2022 May 16;13:881450. doi: 10.3389/fmicb.2022.881450 (PMC9149601; doi:10.3389/fmicb.2022.881450)
Supplement: Supplementary file 1 [file Data_Sheet_1.docx]

Supplementary Tables

# Tables legend

**TABLE S1** Overview of high throughput sequencing

**TABLE S2** PICRUSt2 function prediction for two soil groups of the level 1 KO groups

**TABLE S3** PICRUSt2 function prediction for two soil groups of the level 2 KO groups

**TABLE S4** Trophic modes annotated by FunGuild for two soil groups

**TABLE S5** Guilds based on trophic modes annotated by FunGuild for two soil groups

**TABLE S1** Overview of high throughput sequencing

| Amplification region | Sample names | Number of reads | | OTU numbers | Good’s coverage |
| --- | --- | --- | --- | --- | --- |
|  |  | Raw reads | Clean reads |  |  |
| 16S | hRZ | 36988±5567a | 32300±3955a | 1480±32a | 0.982536±0.000309b |
|  | dRZ | 38911±8304b | 31895±7755a | 1815±49b | 0.980826±0.000504a |
| ITS2 | hRZ | 56425±12630a | 54244±12148a | 322±5a | 0.997822±0.000206a |
|  | dRZ | 44510±5013a | 42740±4999a | 352±13a | 0.997389±0.000195a |

Note: hRZ = healthy rhizosphere soils, dRZ = diseased rhizosphere soils (M ± SD, n = 3). *P*-values are based on matched samples t-test. Significant values (*P* < 0.05) are denoted as by different small letters.

**TABLE S2** PICRUSt2 function prediction for two soil groups of the level 1 KO groups

| Pathway level 1 | hRZ | dRZ | t value | *P* value |
| --- | --- | --- | --- | --- |
| Metabolism | 22484254±31147015.41 | 2634177±1121365.49 | 1.13 | 0.375209 |
| Genetic Information Processing | 22284809±30817713.74 | 2612731±1112621.05 | 1.13 | 0.374779 |
| Environmental Information Processing | 23131512±32090316.87 | 2740128±1176123.31 | 1.13 | 0.376194 |
| Cellular Processes | 20387165±28085605.39 | 2364384±980400.45 | 1.14 | 0.374016 |
| Human Diseases | 21817531±30127895.26 | 2643427±1120682.16 | 1.13 | 0.376351 |
| Organismal Systems | 21562127±29796601.39 | 2598140±1103433.22 | 1.13 | 0.376431 |

Note: hRZ = healthy rhizosphere soils, dRZ = diseased rhizosphere soils (M ± SD, n = 3). *P*-values are based on matched samples t-test. Significant values (*P* < 0.05) are denoted as by different small letters.

**TABLE S3** PICRUSt2 function prediction for two soil groups of the level 2 KO groups

| Pathway level2 | hRZ | dRZ | t value | *P* value |
| --- | --- | --- | --- | --- |
| Cellular community - prokaryotes | 1847606±49757.4 | 1671186±116316.78 | 2.57 | 0.1239 |
| Cell motility | 842301±29507.11 | 775617±62891.57 | 1.65 | 0.240875 |
| Cell growth and death | 610823±21116.62 | 604076±41608.46 | 0.32 | 0.780963 |
| Transport and catabolism | 245365±5274.59 | 229572±5424.12 | 2.88 | 0.102168 |
| Membrane transport | 2171802±71989.42 | 1929655±146367.41 | 3.07 | 0.09168 |
| Signal transduction | 1972734±58381.07 | 1885839±118247.05 | 1.22 | 0.346623 |
| Signaling molecules and interaction | 60±8.07 | 150±55.43 | -2.52 | 0.127635 |
| Translation | 2128305±26784.02 | 2085758±49516.21 | 1.81 | 0.211661 |
| Replication and repair | 1669517±28867.42 | 1633793±52445.17 | 1.21 | 0.351151 |
| Folding, sorting and degradation | 974173±5420.27 | 959029±9964.28 | 2.28 | 0.149807 |
| Transcription | 151518±12275.32 | 147230±23901.69 | 0.35 | 0.759657 |
| Drug resistance: antimicrobial | 557000±25567.59 | 541420±50207.24 | 0.57 | 0.623893 |
| Infectious disease: bacterial | 527972±13107.67 | 527451±25812.32 | 0.04 | 0.970997 |
| Cancer: overview | 432069±6674.97 | 413047±21232.77 | 1.54 | 0.264434 |
| Neurodegenerative disease | 380434±1437.53 | 377708±9758.53 | 0.47 | 0.686591 |
| Infectious disease: viral | 277662±8001.87 | 289546±13076.28 | -2.69 | 0.114537 |
| Drug resistance: antineoplastic | 224590±4491.42 | 218988±12835.49 | 0.87 | 0.478183 |
| Endocrine and metabolic disease | 198229±3287.23 | 190933±8350.04 | 1.52 | 0.26885 |
| Cardiovascular disease | 183084±1926.73 | 178569±7790.27 | 1.11 | 0.382968 |
| Cancer: specific types | 166310±4678.73 | 164177±13236.72 | 0.31 | 0.785039 |
| Infectious disease: parasitic | 56824±1194.98 | 59608±1846.48 | -2.93 | 0.099163 |
| Substance dependence | 26193±2158.33 | 25496±2582.67 | 0.57 | 0.625189 |
| Immune disease | 23346±1458.34 | 19868±1570.25 | 2.77 | 0.10903 |
| Global and overview maps | 30391324±581226.89 | 28523200±982365.97 | 3.02 | 0.094258 |
| Carbohydrate metabolism | 6683329±174293.3 | 6149380±263982.35 | 3.09 | 0.090662 |
| Amino acid metabolism | 6079539±114006.22 | 5696193±199443.7 | 2.98 | 0.096831 |
| Energy metabolism | 3373818±42524.38 | 3224347±78939.15 | 3.01 | 0.094801 |
| Metabolism of cofactors and vitamins | 3218223±33834.75 | 3066432±66656.44 | 3.73 | 0.064853 |
| Nucleotide metabolism | 1690542±21932.57 | 1614428±30873.73 | 3.74 | 0.064643 |
| Xenobiotics biodegradation and metabolism | 1718189±36147.92 | 1533708±86919.49 | 3.29 | 0.081221 |
| Lipid metabolism | 1647346±62834.14 | 1533474±103760.78 | 1.72 | 0.227402 |
| Metabolism of other amino acids | 1189758±40106.19 | 1101925±71652.24 | 1.94 | 0.191474 |
| Biosynthesis of other secondary metabolites | 1167687±33660.32 | 1101323±48563.21 | 2.27 | 0.151053 |
| Glycan biosynthesis and metabolism | 891944±45799.45 | 846699±71353.42 | 1.08 | 0.394093 |
| Metabolism of terpenoids and polyketides | 780768±21148.68 | 734260±35991.34 | 2.19 | 0.160003 |
| Endocrine system | 481404±13505.68 | 446650±23514.92 | 2.22 | 0.156621 |
| Aging | 345096±9624.65 | 335419±21124.84 | 0.77 | 0.522056 |
| Environmental adaptation | 217921±3945.26 | 214063±10607.89 | 0.55 | 0.640022 |
| Nervous system | 119590±3361.67 | 111526±4588.42 | 3.49 | 0.073219 |
| Immune system | 118003±1115.47 | 111375±2778.62 | 5.68 | 0.029641 |
| Circulatory system | 38365±646.35 | 37022±1775.06 | 1.22 | 0.346724 |
| Excretory system | 28445±251.4 | 25052±237.22 | 12.51 | 0.006332 |
| Digestive system | 21628±1782.76 | 20244±1862.4 | 1.06 | 0.401287 |
| Development and regeneration | 11011±506.9 | 11178±1018.46 | -0.26 | 0.817788 |
| Sensory system | 257±56.05 | 124±13.86 | 3.39 | 0.077155 |

Note: hRZ = healthy rhizosphere soils, dRZ = diseased rhizosphere soils (M ± SD, n = 3). *P*-values are based on matched samples t-test. Significant values (*P* < 0.05) are denoted as by different small letters.

**TABLE S4** Trophic modes annotated by FunGuild for two soil groups

| Trophic Mode | hRZ | dRZ | t value | *P* value |
| --- | --- | --- | --- | --- |
| Pathogen-Saprotroph-Symbiotroph | 0.000±0.000 | 0.333±0.577 | 1.00 | 0.422650 |
| Pathotroph | 314.667±54.629 | 274.000±19.000 | 1.56 | 0.258602 |
| Pathotroph-Saprotroph | 57.667±4.041 | 241.333±26.539 | 10.41 | 0.009107 |
| Pathotroph-Saprotroph-Symbiotroph | 6265.333±556.382 | 8073±549.337 | 3.43 | 0.075309 |
| Pathotroph-Symbiotroph | 2606.667±210.842 | 656.333±23.714 | 18.00 | 0.003071 |
| Saprotroph | 19626.000±41.725 | 16201.000±615.929 | 9.03 | 0.012042 |
| Saprotroph-Pathotroph-Symbiotroph | 39.000±4.583 | 17.667±12.662 | 2.20 | 0.158913 |
| Saprotroph-Symbiotroph | 3902.000±155.232 | 3854.333±370.832 | 0.18 | 0.873041 |
| Symbiotroph | 1.667±1.155 | 0.000±0.000 | 2.50 | 0.129612 |
| Unknown | 5743.000±264.456 | 9230.667±893.356 | 5.61 | 0.030387 |

Note: hRZ = healthy rhizosphere soils, dRZ = diseased rhizosphere soils (M ± SD, n = 3). *P*-values are based on matched samples t-test. Significant values (*P* < 0.05) are denoted as by different small letters.

**TABLE S5** Guilds based on trophic modes annotated by FunGuild for two soil groups

| Guild | hRZ | dRZ | t value | *P* value |
| --- | --- | --- | --- | --- |
| Animal Endosymbiont | 24.667±9.504 | 172.333±23.714 | 7.76 | 0.016204 |
| Animal Parasite | 0.667±0.577 | 5.333±5.033 | 1.79 | 0.214919 |
| Animal Pathogen | 5891.333±505.415 | 7515.333±465.491 | 3.46 | 0.074390 |
| Clavicipitaceous Endophyte | 29.667±7.095 | 58.667±3.055 | 5.21 | 0.034940 |
| Dung Saprotroph | 3406.000±93.150 | 3034.667±413.795 | 1.78 | 0.216365 |
| Ectomycorrhizal | 1.00±0.000 | 0.667±0.577 | 1.00 | 0.422650 |
| Endomycorrhizal | 5.667±0.577 | 0.000±0.000 | 17.00 | 0.003442 |
| Endophyte | 1442.333±190.768 | 1687.333±61.655 | 3.03 | 0.093878 |
| Epiphyte | 2566.667±202.804 | 553.000±27.731 | 19.88 | 0.002520 |
| Ericoid Mycorrhizal | 1.333±0.577 | 0.667±0.577 | 2.00 | 0.183503 |
| Fungal Parasite | 438.000±55.507 | 610.000±114.241 | 2.14 | 0.165193 |
| Leaf Saprotroph | 12071.000±168.057 | 11389.000±390.862 | 2.66 | 0.116897 |
| Lichen Parasite | 4.000±3.606 | 5.667±1.528 | 0.56 | 0.630389 |
| Lichenized | 0.000±0.000 | 0.333±0.577 | 1.00 | 0.422650 |
| Orchid Mycorrhizal | 39.000±4.583 | 17.667±12.662 | 2.20 | 0.158913 |
| Plant Saprotroph | 262.667±57.073 | 238.000±7.810 | 0.84 | 0.490442 |
| Soil Saprotroph | 16.667±4.163 | 5.000±3.000 | 3.55 | 0.070869 |
| Undefined Saprotroph | 6611.333±261.338 | 4022.000±219.447 | 14.28 | 0.004869 |
| unknown | 5743.333±264.791 | 9231.333±892.956 | 5.61 | 0.030353 |
| Wood Saprotroph | 0.667±1.155 | 1.667±0.577 | 1.00 | 0.422650 |

Note: hRZ = healthy rhizosphere soils, dRZ = diseased rhizosphere soils (M ± SD, n = 3). *P*-values are based on matched samples t-test. Significant values (*P* < 0.05) are denoted as by different small letters.
