# Supplementary figures and images for: Comparative Analysis of the Microbial Community Structures Between Healthy and Anthracnose-Infected Strawberry Rhizosphere Soils Using Illumina Sequencing Technology in Yunnan Province, Southwest of China
Source: Front Microbiol. 2022 May 16;13:881450. doi: 10.3389/fmicb.2022.881450 (PMC9149601; doi:10.3389/fmicb.2022.881450)

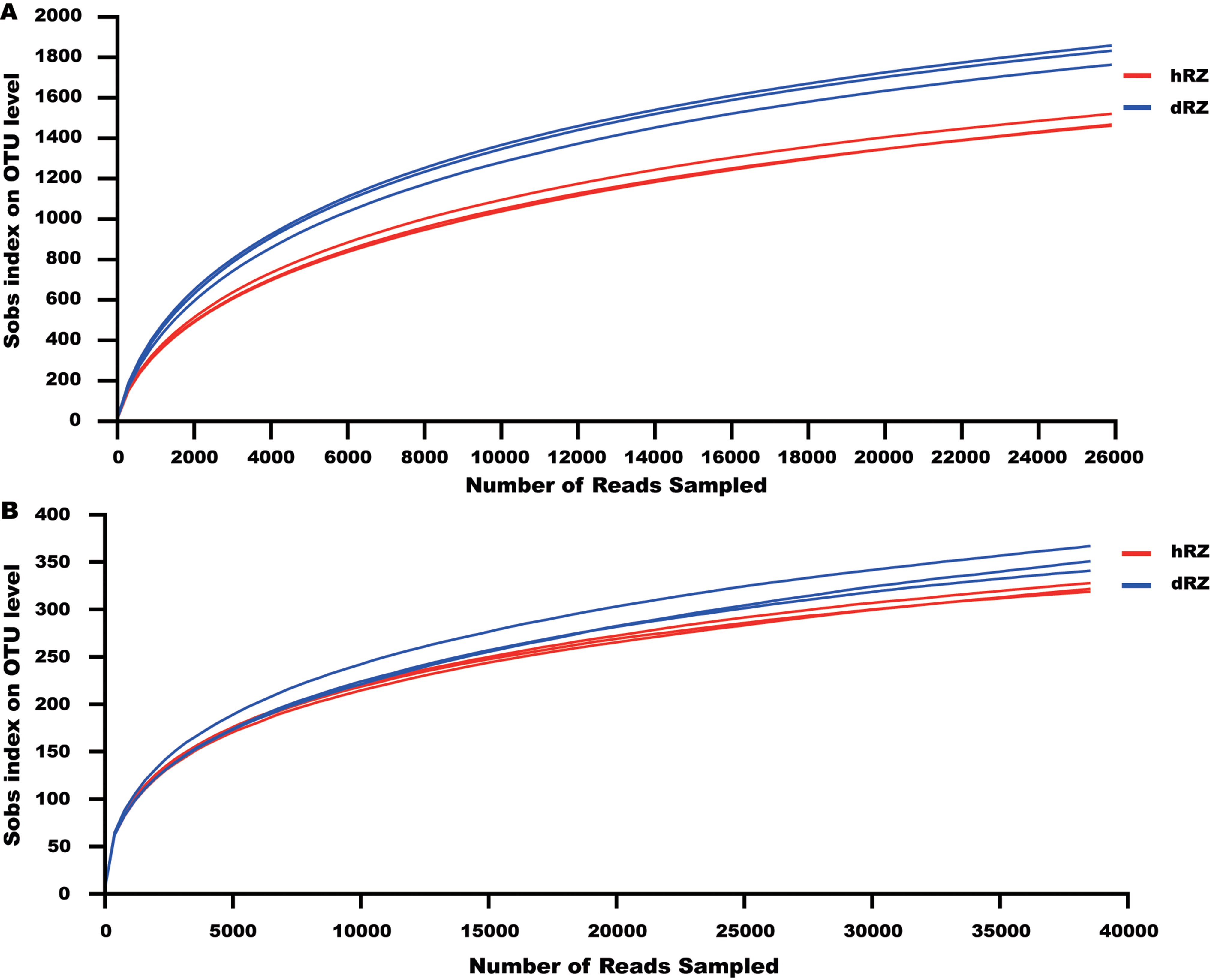

Supplement: Supplementary file 2 [file Image_1.tif]

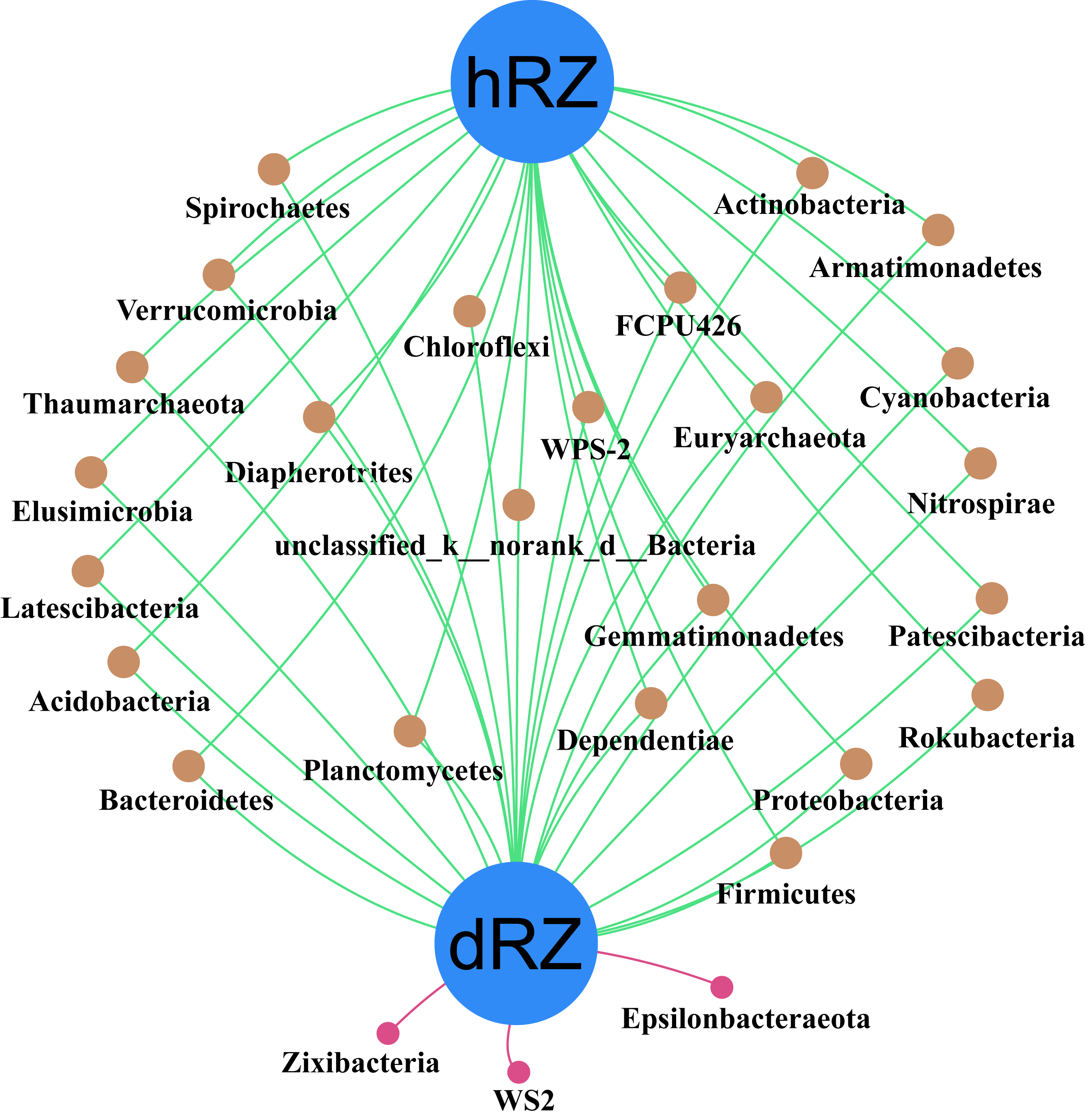

Supplement: Supplementary file 3 [file Image_2.tif]

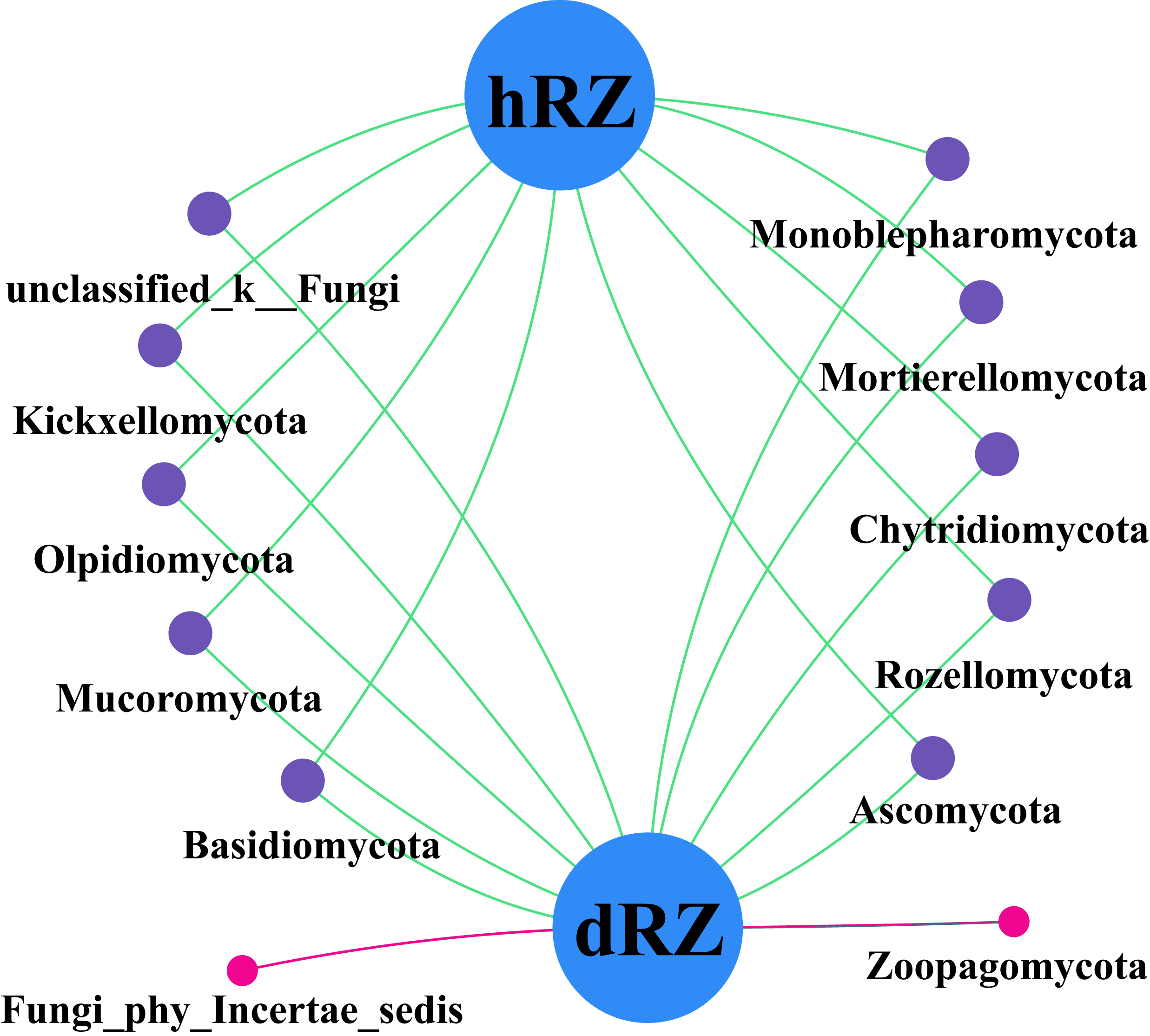

Supplement: Supplementary file 4 [file Image_3.tif]

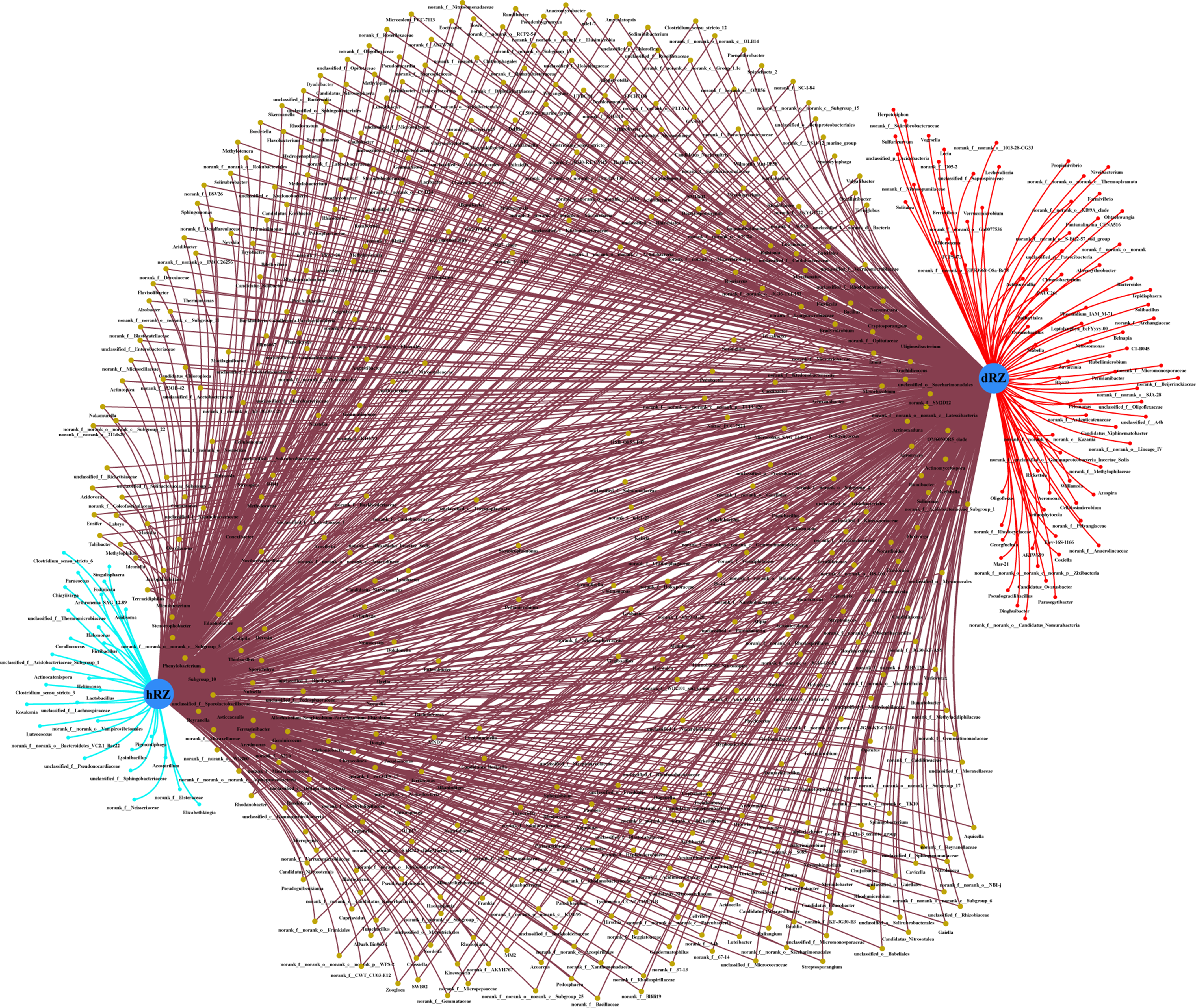

Supplement: Supplementary file 5 [file Image_4.tif]

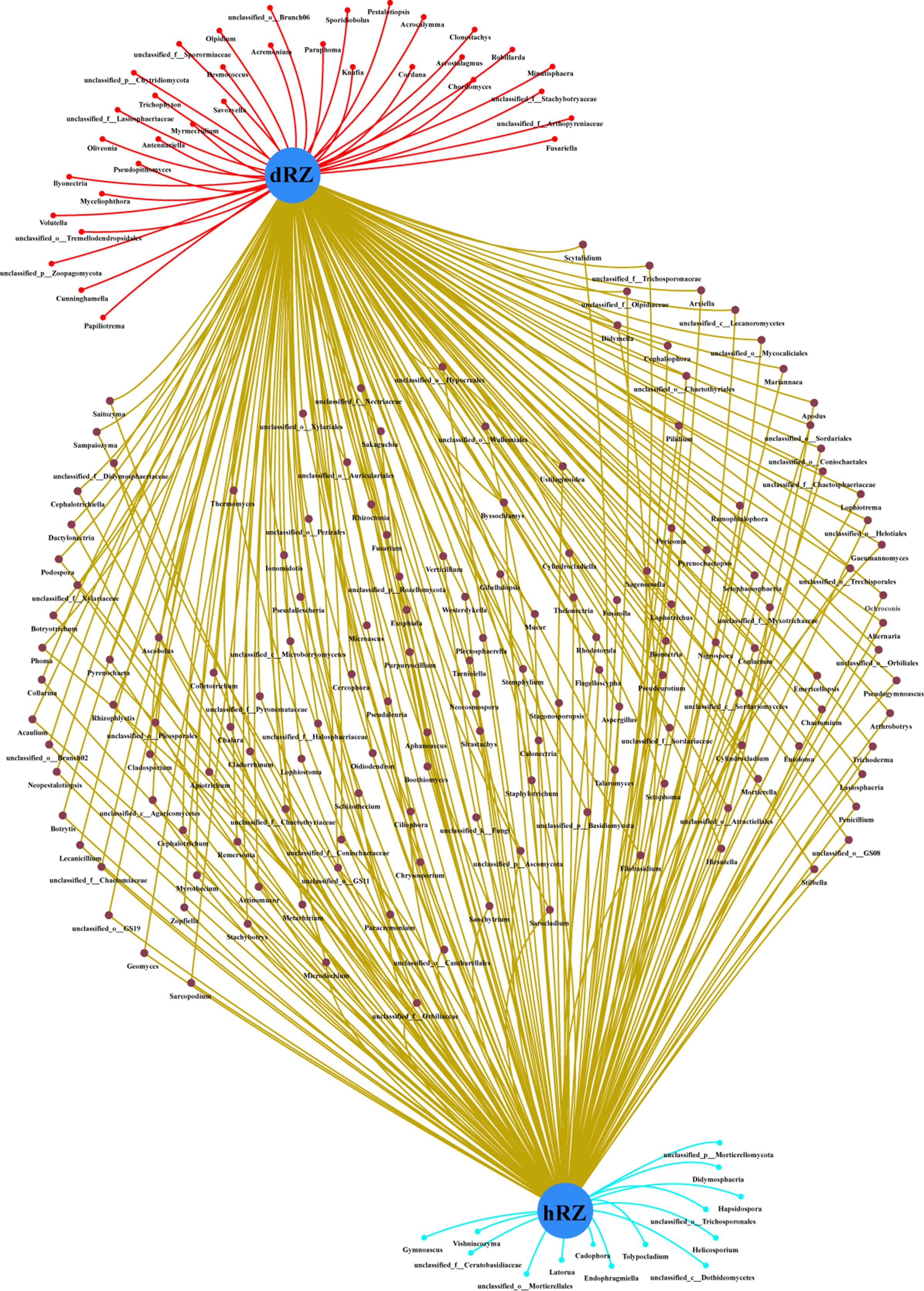

Supplement: Supplementary file 6 [file Image_5.tif]
